# Intermittent Hypoxia Selects for Genotypes and Phenotypes That Increase Survival, Invasion, and Therapy Resistance

**DOI:** 10.1371/journal.pone.0120958

**Published:** 2015-03-26

**Authors:** Daniel Verduzco, Mark Lloyd, Liping Xu, Arig Ibrahim-Hashim, Yoganand Balagurunathan, Robert A. Gatenby, Robert J. Gillies

**Affiliations:** 1 Department of Cancer Imaging and Metabolism, H. Lee Moffitt Cancer Center & Research Institute, Tampa, Florida, United States of America; 2 Analytic Microscopy Core, H. Lee Moffitt Cancer Center & Research Institute, Tampa, Florida, United States of America; 3 Department of Radiology, H. Lee Moffitt Cancer Center & Research Institute, Tampa, Florida, United States of America

## Abstract

Hypoxia in tumors correlates with greater risk of metastases, increased invasiveness, and resistance to systemic and radiation therapy. The evolutionary dynamics that links specific adaptations to hypoxia with these observed tumor properties have not been well investigated. While some tumor populations may experience fixed hypoxia, cyclical and stochastic transitions from normoxia to hypoxia are commonly observed *in vivo*. Although some phenotypic adaptations to this cyclic hypoxia are likely reversible, we hypothesize that some adaptations may become fixed through mutations promoted by hypoxia-induced genomic instability. Here we seek to identify genetic alterations and corresponding stable phenotypes that emerge following cyclic hypoxia. Although these changes may originate as adaptations to this specific environmental stress, their fixation in the tumor genome may result in their observation in tumors from regions of normoxia, a condition known as *pseudohypoxia*. We exposed several epithelial cell lines to 50 cycles of hypoxia-normoxia, followed by culture in normoxia over a period of several months. Molecular analyses demonstrated permanent changes in expression of several oncogenes and tumor-suppressors, including p53, E-cadherin, and Hif-1α. These changes were associated with increased resistance to multiple cytotoxins, increased survival in hypoxia and increased anchorage-independent growth. These results suggest cycles of hypoxia encountered in early cancers can select for specific and stable genotypic and phenotypic properties that persist even in normoxic conditions, which may promote tumor progression and resistance to therapy.

## Introduction

As cancer cells progress, they undergo an evolutionary process during which genetic or epigenetic changes accumulate leading to progressively aggressive phenotypes. The common phenotypes associated with malignant disease are represented by the “hallmarks of cancer”, and include changes in proliferation, apoptosis resistance, and metabolism [1]. We have proposed that the emergence of these hallmarks results from selection by factors, such as hypoxia, that are altered as the microenvironment changes during carcinogenesis [2]. Tumor hypoxia is associated with radio-resistance and drug resistance [3–6]. This is relevant because drug resistance is the major cause of treatment failure for cancers and this may result from minor populations of drug resistant cells that are present within tumors prior to drug treatment [7]. Exposure to chemotherapeutics selects for outgrowth of these already pre-existing drug resistant cell populations [8]. Importantly, we hypothesize that some of the phenotypic alterations induced by hypoxia can become hardwired so that they are expressed constitutively, even under normoxic conditions, a state known as pseudohypoxia [9, 10].

*In vitro* studies have shown that hypoxia selects for cells carrying mutations in p53 such that minor populations engineered to carry nonfunctional alleles of *TP53* eventually overtake populations wild-type for *TP53* [11], which can lead to apoptosis resistance. Notably, it has been shown that hypoxia leads to a downregulation of DNA damage repair pathways, and increased DNA damage upon re-oxygenation [12]. Hence, hypoxia can not only induce genetic changes, but it may select for cells that are resistant to apoptosis and thus able to survive in the face of accumulating genetic changes. As a tumor is composed of variable adaptive landscapes caused by an imbalance of blood supply, adaptation of different cancer cell populations to these different microenvironments can lead to heterogeneous populations of cancer cells within a single tumor, as has been detected by multiple biopsies from single patient tumors [13–15]. Furthermore, intermittent hypoxia and chronic hypoxia are generally regarded as distinct phenomena [16], leading to differential effects on tissues and therefore different therapeutic consequences. This heterogeneous genetics leads to increased complexity not only for treatment regimens, but also in the outcomes of therapy.

In this study, we report the acquisition of stable phenotypes in response to selection by intermittent hypoxia. Notably, these adaptations confer resistance to chemotherapeutics to which the cells were otherwise naïve. Altered expression of p53, E-cadherin, and HIF-1α underlie these phenotypic changes and, in some cases, these expression changes were associated with chromosomal loss. Thus, we propose that intermittent hypoxia during early carcinogenesis leads to somatic evolution resulting in drug resistance and increased aggressiveness. Furthermore, we show that some of these changes can occur consistently across different cell types.

## Results

**Fig 1:**
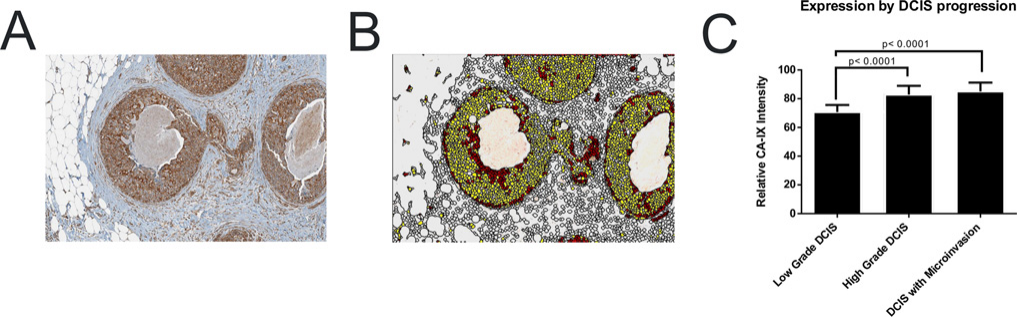
CA-IX is expressed in pseudohypoxic tissue. 200x magnification image of ductal carcinoma of the breast with microinvasion A) stained against CA-IX and B) segmented into individual cells and color coded by quantitative intensity where white is excluded as stroma, yellow is weak, orange is moderate and red is strong staining intensity. C) Bar graph of CA-IX intensity per lesion type from low grade DCIS to high grade DCIS and DCIS with microinvasion.

During early carcinogenesis, when neoplastic cells are confined within ducts, areas of severe hypoxia are evident in the periluminal regions. This has been inferred from immunohistochemistry (IHC) of patient tissue for the Hypoxia-Inducible Factor (HIF) clients, CA-IX (Fig. 1) and GLUT-1 (S1 Fig.) [17, 18]. Periluminal hypoxia develops because the blood supply is restricted to the stroma and the maximum diffusion distance of oxygen in tissues is <200 microns [19]. Further, due to inconsistencies in blood flow, there is mathematical and empirical evidence that this hypoxia is intermittent, with periodicities from minutes [20–22] to days [23].

Glut-1 and CA-IX expression are induced by hypoxia via HIF, which is stabilized under hypoxia. These factors are often constitutively expressed in malignant tissues, even in the presence of oxygen, a condition known as “pseudohypoxia”. Fig. 1A shows a section of human Ductal Carcinoma in situ (DCIS) stained for CA-IX. Using pattern recognition technology (Tissue Studio v3.0; Definiens; Munich, Germany), individual cells in stroma and DCIS were segmented and the CA-IX staining intensity was classified as either strong, moderate, or weak, corresponding to pathologist’s classification of 3+, 2+ or 1+, respectively. These were color coded red, yellow and white, respectively in Fig. 1B, which shows the presence of strongly positive CA-IX staining in the peri-luminal zone consistent with hypoxia, and the peripheral zone, which may be evidence for pseudohypoxia. Measurements across multiple tumors indicate that CA-IX is a marker for increased grade (Fig. 1C) and poorer prognosis.

**Fig 2:**
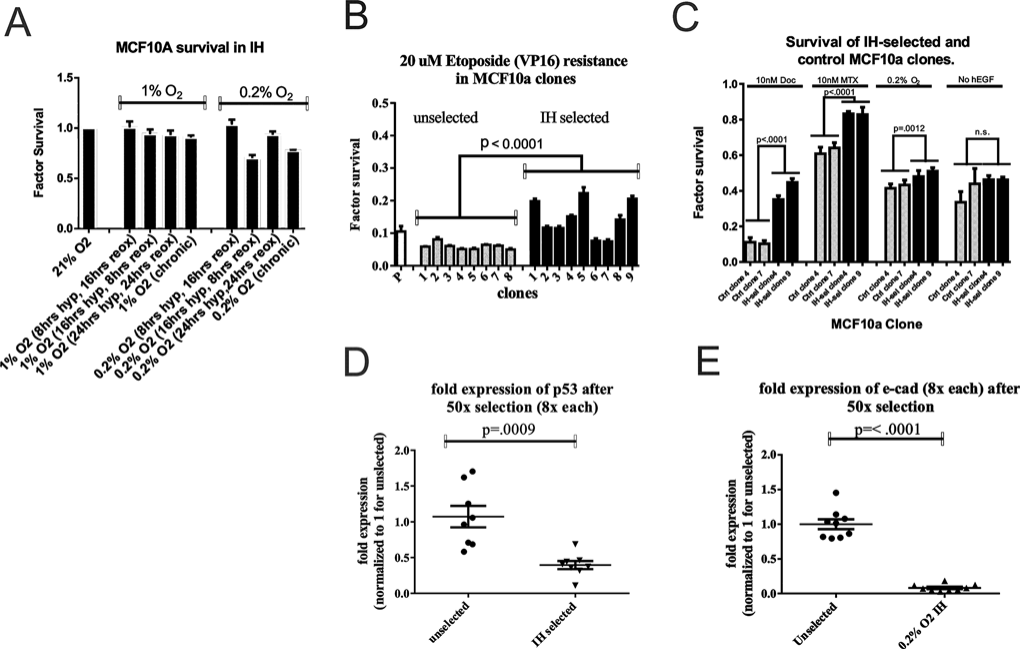
Selection of MCF10A cells by intermittent hypoxia (IH) induces drug resistance and reduced expression of p53 and E-cadherin. (A) Multiple intermittent hypoxia regimens were tested at 1%O_2_ and 0.2% O_2_ over 6 days. Factor survival is expressed as compared to cells grown in parallel in normoxia (21%O_2_). MCF10A cells undergoing repetitive cycles of 16 hours hypoxia followed by 8 hrs reoxygenation exhibited the most cell death. (B) MCF10A cells were cultured in 50 cycles of IH in the selective regimen described in (A), and individual cells were isolated to be raised as clones, and then passaged for 2 months in normoxia. These clonal populations were tested for etoposide resistance. Heterogeneity in the IH-selected clones was detected, while a significant overall increase in etoposide resistance was measured. (C) MCF10A IH-selected clones 4 and 9 were further tested for resistance to multiple cytotoxic conditions, including treatment to the microtubule stabilizing docetaxel, the folate metabolism inhibitor methotrexate, as well as hypoxia and reduction of growth factors. Intermittent hypoxia exhibited increased survival to all of these conditions except for survival to growth factor removal. (D,E) Intermittent hypoxia exhibits changes in the expression of p53 and E-cadherin. Quantitative Real-Time PCR was performed on each IH-selected and passage control clone. Reduced *p53* and E-cadherin mRNA expression levels were detected in IH-selected control clones. Factor expression is relative to the average of control clones.

Changes in oxygen tensions have been shown to inflict genotoxic stress upon cells and inhibit cell proliferation [24–26]. Long exposures to conditions of <1% O_2_ can lead to cell death via apoptosis in a dose-dependent manner [27, 28]. Additionally, re-oxygenation can cause the creation of radical oxygen species (ROS) that can lead to DNA strand breaks [12]. The rate of evolution is related to both phenotypic heterogeneity and selection strength, and thus we propose that acute or intermittent hypoxia (IH) may lead to somatic evolution driven by both of these components [29]. To determine the strength of selection, we subjected MCF10A breast epithelial cells (Fig. 2A), MDA-MB-231 metastatic breast cancer, RKO colorectal cancer cells, SU86.86 metastatic pancreatic cancer cells, and MCF10.DCIS cells (S2 Fig.) to different regimens of intermittent or chronic hypoxia. Cell numbers were determined after six-day growth periods with circadian oxygenation periods between normoxia and hypoxia with 0.2% or 1% oxygen. Cycles ranged from 24hr hypoxia-24 hr reoxygenation, 16 hrs hypoxia-8 hrs reoxygenation, or 8 hrs hypoxia-16 hr reoxygenation, chronic hypoxia or normoxia. For all cells tested, subjecting them to 16 hrs 0.2% hypoxia + 8 hrs normoxia demonstrated the greatest amount of cell death of the intermittent hypoxia regimens tested, which we equate to selection pressure. This periodicity was consistent with others [30].

To test the long-term effects of intermittent hypoxia on breast epithelia, we subjected MCF10A, transformed non-invasive MCF10.DCIS, and invasive metastatic MDA-MB-231 cells to 50 rounds of intermittent hypoxia with 16 hours of 0.2% hypoxia followed by 8 hours of normoxia. Following this selection, we isolated and cultured clonal populations raised from single cell suspensions, which we refer to as simply ‘clones’. Clonal assays were used because, although we expect hypoxic selection will lead to population drift, we also expect it to generate genetic and phenotypic heterogeneity across the population, which would be evident in dispersed clones. Furthermore, from an evolutionary dynamic perspective, the presence of even a minor population could have significance under the appropriate selection conditions, and that these would not be observed in polyclonal analyses. For each cell line tested, we isolated up to 9 each of intermittent hypoxia (IH)-selected clones and clones from passage-controlled cells that were never exposed to hypoxia. All clones were passaged for at least 2 months in normoxia before any characterization was performed to ensure that any phenotypic changes were fixed and not due to transient adaptations to the environment.

Initially, we tested MCF10A, MCF10.DCIS, and MDA-MB-231 clones for survival against 20uM etoposide. Etoposide (VP-16) is a topoisomerase inhibitor that is believed to kill cells exclusively via apoptosis. Hypoxia has previously been shown to induce resistance to etoposide-mediated cell death in a p53-dependent manner [31, 32]. As shown in Fig. 2B, IH-selected clones were heterogeneous in their response to etoposide, with some (i.e. 1,5,9) being highly resistant, whereas others (i.e. 6,7) were as sensitive as the controls. Notably, there was little heterogeneity among the control clones in their sensitivity to etoposide. Despite the heterogeneity of phenotypes, as a group the IH-selected cells were significantly more resistant to etoposide killing than were the non-selected variants (p<0.0001). In contrast, both IH-selected and non-selected MCF10.DCIS cells were resistant to etoposide and both exhibited high degrees of heterogeneity and there was no difference between the selected and control groups (S3A Fig.). This is an interesting observation that suggests that these cells have already been selected for apoptosis resistance prior to the commencement of this experiment. The more malignant clones from MDA-MB-231 cells were interesting in that, although the unselected clones were uniformly resistant, similar to the IH-selected MCF10A clones 1,5 and 9; the IH-selected MDA-MB-231 clones were more heterogeneous with some (i.e. clone 7) being extremely resistant S3B Fig.). As a group, the IH-selected MDA-MB-231 clones were more resistant than were the unselected controls (P<0.0001).

We subsequently examined two MCF10A IH-selected clones (clones 4 and 9) and two control clones (clones 4 and 7) in more detail. IH clones 4 and 9 exhibited the greatest survival to 10 μM etoposide, while control clones 4 and 7 exhibited the least and most resistance, respectively, among the control clones (data not shown). In addition to survival against etoposide, the IH-selected clones demonstrated increased survival when treated with methotrexate and docetaxel (Fig. 2C). Methotrexate survival canonically relies on amplified *DHFR*. This gene amplification event as well as survival to methotrexate has been reported to occur after intermittent hypoxia [33]. We also tested for survival against the anti-mitotic docetaxel, which is a commonly used breast cancer drug. The two IH-selected clones demonstrated resistance to 10nM docetaxel. To test whether these clones exhibited general survival benefits to microenvironmental stressors as well, we measured survival with reduced growth factors or chronic hypoxia (Fig. 2C). Each of these conditions may occur during intraductal carcinogenesis. Of the clones tested, each IH-selected clone exhibited increased survival in response to hypoxia, but did not have reduced growth factor independence. Overall, these results indicate that these cells have a general resistance to apoptosis. However they are not independent of growth factors, which is a distinct Hallmark of Cancer mechanism [34].

To investigate whether these phenotypic changes were associated with underlying genomic changes, we utilized the Nanostring nCounter analysis system for karyotyping (Nanostring Technologies, Seattle, Washington) to compare the genomes of IH-selected clone 4 to control clone 7. These results showed several regions throughout the genome that had lost one allele, and there were very few amplification events. In particular, karyotyping showed loss of a portion of 17p that contains TP53 and of 16q that contains CDH1, the gene that encodes the tumor suppressor, E-cadherin [35]. Traditional (Giemsa) karyotyping (U Minnesota Cytology core) did not identify these specific alterations, but did confirm that these cells were still karyotypically similar to MCF10A cells [36].

Because the NanoString data suggested loss of chromosomal elements close to the P53 and CDH1 genes, we then performed quantitative real-time qPCR to determine the status of E-cadherin and p53 genes across all clones. Notably, the reduction of E-cadherin and of p53 transcripts were observed across all IH-selected clones (Fig. 2D-E). Interestingly, some unselected clones were observed to have low p53 transcripts, suggesting that a reduced p53 phenotype may have pre-existed in the population prior to selection. Conversely, all control clones had high levels of E-cadherin mRNA. To test whether this is consistent, we repeated our experiment on cells treated with IH daily for two weeks, for a total of 15 hypoxia-reoxygenation cycles. Cells were initially seeded at 10,000 cells/well with 8 wells per condition, and mRNA was isolated at the end of the experiment. These selected cells also exhibited reduced E-cadherin and p53 expression (S4 Fig.), indicating a consistent mechanism for loss of the expression of these genes. Conversely, cells treated with low pH and low glucose, conditions which are also selective [37], did not exhibit reduced p53 (S4 Fig.)

Further, we tested whether the observed changes were due to intermittent hypoxia or whether they may be induced by chronic hypoxia. We cultured MCF10A cells in chronic hypoxia (0.2% O_2_) conditions for 2 months, followed by 2 months of normoxic growth during which clonal selection was performed. We measured the levels of E-cadherin and p53 in 10 clones isolated in this way and detected no significant difference (S5 Fig.). Additionally, we measured survival of these cells when treated with 20μM etoposide, 10nM docetaxel, and 3 days of hypoxic growth, and we detected no significant change in survival under any of these conditions (S6 Fig.).

As GAPDH was used to normalize RT-PCR and western data, we confirmed that GAPDH levels were not affected by selection by comparing GAPDH expression levels to that of actin. We detected no significant difference either between our parental controls and parental IH-selected cells, or between parental controls and IH-selected clones 4 and 9 (S7 Fig.).

**Fig 3:**
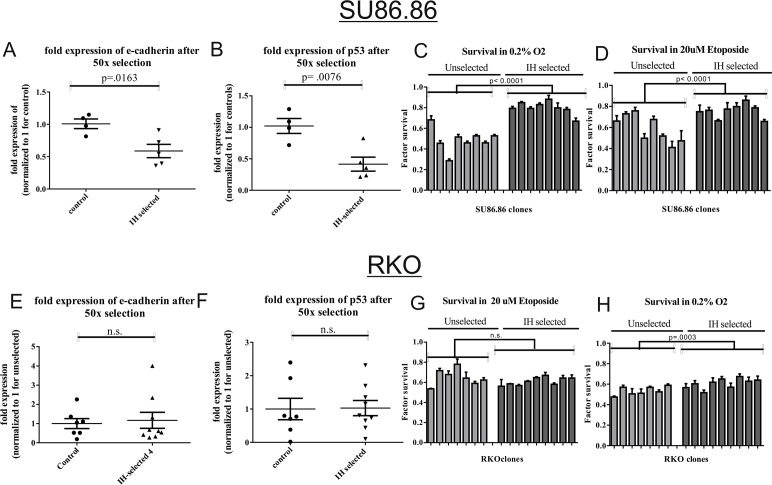
SU86.86 cells exhibit phenotypic and genotypic changes following selection by IH. SU86.86 cells were treated with IH for 50 cycles, had clonal populations raised, and grown in normoxia for 2 months. A significant decrease in expression of E-cadherin (A) and p53 (B) was detected by quantitative Real-Time PCR. IH-selected SU86.86 clones also exhibited an increase in resistance to etoposide (C) and hypoxia (D).Results of selection of RKO cells following IH-selection (E-H). We do not detect any changes in p53 or E-cadherin expression within clones of RKO IH-selected (E,F). Furthermore, these cells did not exhibit resistance to etoposide (G), nor hypoxia (H).

Our data indicate that intermittent hypoxia selects for loss of E-cadherin and p53 in MCF10A cells. To test whether this phenomenon is observed in other cells types, we subjected SU86.86 pancreatic carcinoma cells to 50 rounds of intermittent hypoxia to test whether E-cadherin expression would be lost. These cells were chosen because they carry functional alleles of CDH1, but have mutant p53. We hypothesized that following 50 rounds of intermittent hypoxia, that SU86.86 clones would exhibit reduced E-cadherin transcripts, but that p53 transcripts would be unaffected. Consistent with this hypothesis, we detected reduced E-cadherin transcripts in multiple SU86.86 clones (Fig. 3A). However, we also detected reduced p53 transcripts, indicating a general mechanism of reduced p53 expression following IH (Fig. 3B). The G245S p53 mutation in this cell line is generally considered to be inactive [38, 39]. Consistent with its p53 status, clones of unselected SU86.86 cells were highly resistant to etoposide and hypoxia. However, the IH-selected clones were even more resistant (Fig. 3C-D) suggesting the presence of some residual pro-apoptotic activity in these cells. Intermittent hypoxic selected RKO colorectal carcinoma cells are E-cadherin null and p53 wild-type, and these were also subjected to 50X cycles of intermittent hypoxia to test the hypothesis that this would select for loss of p53. Notably, these selected cells do not exhibit differences in either p53 or E-cadherin expression compared to passage-controlled cells (Fig. 3E-F). Despite this, RKO cells exhibited increased resistance to hypoxia, but not to etoposide, suggesting the presence of separate mechanisms conferring hypoxia resistance in these cells.

**Fig 4:**
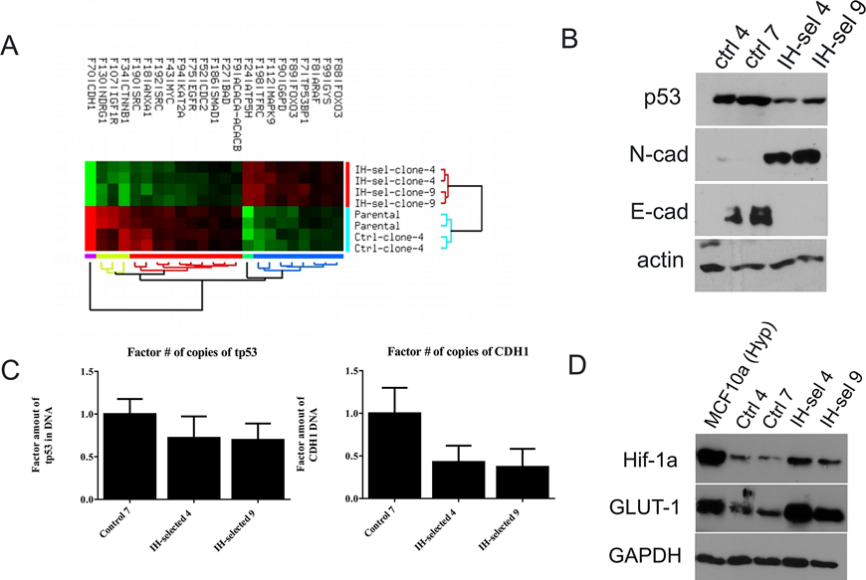
Differential protein levels and phenotypes are exhibited among MCF10A cells following selection through IH. (A) Analysis of protein levels was performed using the Reverse Phase Protein Array (RPPA) on the parental MCF10A line, passaged control clone 7, IH-selected clone 4, and IH-selected clone 9. Each clone was submitted in duplicate. Hierarchical clustering of significant (FDR ≤ 0.05) protein markers in control versus IH-selected samples is shown. The clustering was carried out with Euclidean distance metric with average linkage, and the data was median normalized and log2 transformed. The clustering was arbitrarily stopped at 2 and 5 groups for the samples and features respectively. (B) Western blotting analysis of p53 and E-cadherin protein. p53 protein is reduced by in IH-selected clones. E-cadherin is completely lost following selection by IH, and N-cadherin protein is detectable, indicating EMT in IH-selected clones. (C) Quantitative RT-PCR was performed on DNA levels of the *P53* and *E-CAD* loci. A 0.5 fold reduction in the number of copies of *P53* and *E-CAD* indicates a loss of one allele of each. (D) Western blotting against HIF-1α and GLUT-1 indicates constitutive stabilization of HIF-1α and its downstream effector GLUT-1 in IH-selected cells. MCF10A cells cultured in hypoxia are used as a positive control.

To measure multiple cancer-related proteins in a higher throughput manner, we submitted protein samples of the parental MCF10A cells as well as a MCF10A passage control (control-7) and two intermittent hypoxic clones (IH-sel 4 and IH-sel 9) to the M.D. Anderson Reverse Phase Protein Array (RPPA) facility. The results were normalized using linear methods based on internal controls, median centered and log2 transformed prior to analysis. Student’s T-test (Welch) was performed to find significant markers between control and IH-selected groups. In order to control the family-wise multiple testing errors, the False Discovery Rate (FDR) was computed [40, 41]. There were 26 protein markers that were found to be significant (*p* ≤ 0.05, FDR *q* ≤ 0.05). These differentially expressed markers were used in the clustering analysis to illustrate the expression difference (Fig. 4A). A Principal Components Analysis (PCA) revealed clear separation between hypoxic and normal samples using 26 significant protein markers (Table 1). As expected, the two intermittent hypoxic selected clones were more closely related than were the normoxic control clone and the parental line. The RPPA assay also confirmed reduced E-cadherin and p53 protein levels, but also changes in the levels of other proteins, such as reduced c-myc and annexin-I.

**Table 1:** Details of the significant protein markers with FDR and fold change (log of Control to IH-selected group).

Protein Name	Ttest (P-value)	FDR (Q-value)	Fold Change (Log2 of Norm/Hypoxic)
Src_pY527	0.000004	0.000471	0.449689
E-Cadherin	0.000003	0.000573	3.028638
beta-Catenin	0.000014	0.000588	1.207492
FOXO3a	0.000009	0.000616	-0.25395
c-Myc	0.000019	0.000669	0.553257
GYS	0.000013	0.000715	-0.30252
JNK2	0.000097	0.002604	-0.60568
TFRC	0.00009	0.002751	-0.89242
EGFR_pY1068	0.000265	0.00631	0.340197
FOXO3a_pS318_S321	0.00074	0.014406	-0.44514
GCN5L2	0.000711	0.015225	0.369197
CDK1	0.000978	0.01744	0.310195
NDRG1_pT346	0.001094	0.018004	0.977278
Src	0.001199	0.018324	0.674909
G6PD	0.001674	0.023889	-0.40407
Bad_pS112	0.0028	0.033295	0.138613
Smad1	0.002656	0.033439	0.161755
ATP5H	0.002533	0.033884	-1.2394
Annexin_I	0.003035	0.03418	0.784935
53BP1	0.003943	0.040185	-0.37454
IGF1R	0.003773	0.040371	0.774288
A-Raf	0.004518	0.043949	-0.17145
ACC_pS79	0.004763	0.044314	0.241103
GSK3-alpha-beta_pS21_S9	0.005928	0.050739	-0.18914
CDKN2A_p16INK4a	0.005704	0.050865	-0.11895
GAPDH	0.00657	0.054079	-0.38184

To confirm these data and our RT-PCR data, protein levels for p53, N-cadherin, and E-cadherin were measured by Western blot, and reduced p53 protein and E-cadherin protein levels were observed (Fig. 4B). The epithelial-to-mesenchymal transition (EMT) is the process by which cells lose their epithelial characteristics and become more migratory. During EMT, N-cadherin protein is upregulated while E-cadherin is lost. Also shown by western blot, IH-selected clones exhibit increased N-cadherin indicating the occurrence of EMT following selection by IH. This is consistent with others who have proposed that hypoxia can induce EMT [42, 43]. However, the difference in the present study is that the genotype/phenotype is stable, as expression levels were determined following 2 months of normoxic growth following the 50 rounds of selection. We measured the DNA content of these cells by performing quantitative RT-PCR on DNA isolated from these cells and utilizing primers derived from intronic regions of these genes [44]. A 2-fold change in expression by this technique indicates a loss of half of the number of the alleles. As expected, and confirming our karyotyping data, we determined that IH-selected cells carried half of the number of copies of both the P53 and CDH1 alleles (Fig. 4C).

As cells exhibit numerous physiological and genetic changes during hypoxia, we explored whether IH-selected cells had fixed any of the phenotypes that occur during hypoxia. These include an induction of the HIF client, CA-IX, as well as increased glycolysis. In control studies, we determined that MCF10A cells upregulated CA-IX under hypoxia compared to normoxia; but that MDA-MB-231 and MCF10.DCIS cells constitutively expressed CA-IX even in normoxic conditions (S8 Fig.). We tested for the expression levels of several hypoxia related proteins within our IH-selected clones (Fig. 4D). HIF-1α is stabilized upon exposure to hypoxia, but is quickly destroyed upon reoxygenation. We measured the expression of HIF-1α and two of its target genes, GLUT-1 and CA-IX in our IH-selected cells, our passaged-control cells, the parental MCF10a, and, as a positive control, the parental MCF10a cells incubated in hypoxia. As expected, MCF10a cells strongly upregulated HIF-1α, GLUT1, and CA-IX under hypoxia. Further, the IH-selected clones, despite being passaged in normoxia for months, exhibited increased HIF-1α and GLUT1 compared to passage controls, indicating the acquisition of the pseudohypoxic phenotype within these cells. Additionally, we detected an increase in CA-IX mRNA and protein levels in IH-selected clones relative to passage controls (S9 Fig.).

**Fig 5:**
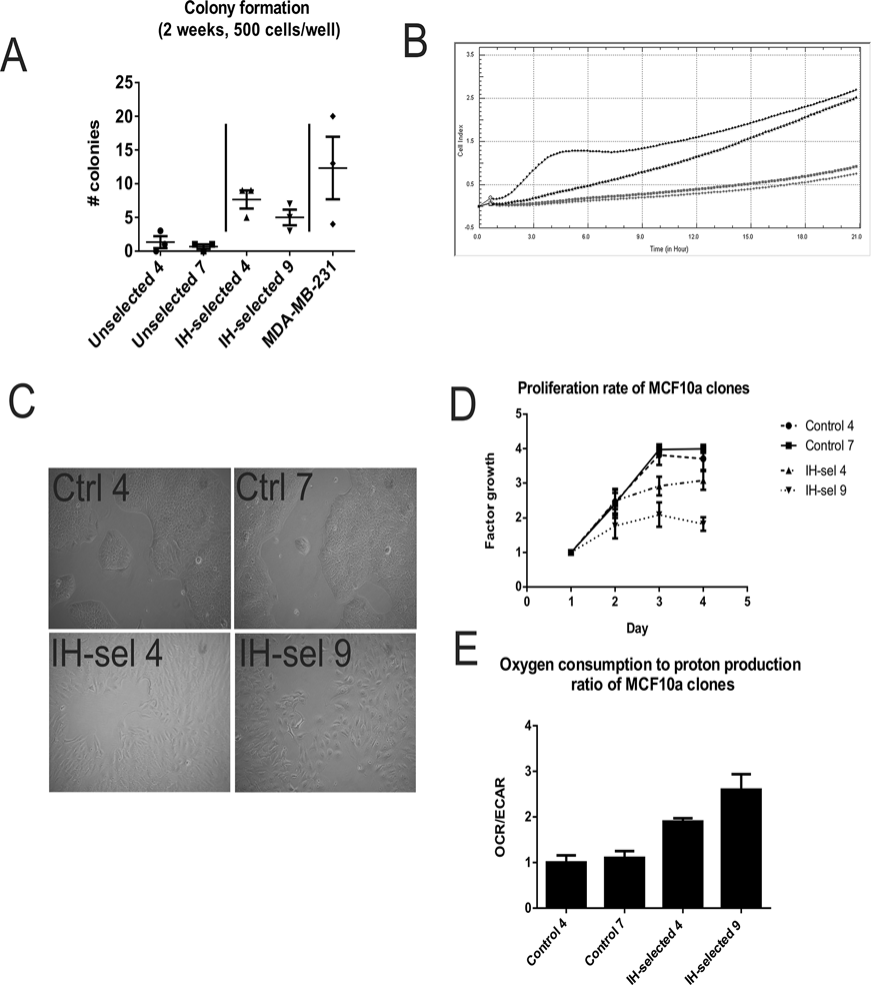
(A) Soft agar colony formation assays were performed on each clone as a measure of cell-adhesion growth. IH-selected clones demonstrated an increase in the number of colonies relative to passage control clones. Metastatic MDA-MB-231 cells were seeded as a positive control. (B) Passage control MCF01A clones (top) and IH-selected clones (bottom) were imaged using brightfield microscopy. While control clones exhibited smooth borders indicative of an epithelial phenotype, IH-selected cells migrated apart from adjacent cells, indicating an increase in migration as well as EMT. (C) As a measure of migratory capacity, passage control MCF10A clones (in grey) and IH-selected MCF10A (in black) were seeded within an xCELLIGENCE CIM plate, which measures in real time the number of cells passing through a barrier from a low-serum well to high-serum well. Measurements taken over the span of 24 hours indicates IH-selected clones have increased migratory rates (D) Proliferation rates of each of the four clones. Cell counts were performed daily. IH-selected clones exhibited reduced proliferation relative to passage control clones. (E) Oxygen consumption and proton production (a proxy for glycolysis) were measured for each clone with the Seahorse XF Analyzer. The ratio of oxygen consumption to proton production was increased in IH-selected clones.

Consistent with loss of E-cadherin, the IH-selected clones exhibited adhesion-free growth in a soft agar colony formation assay (Fig. 5A). Consistent with this behavior, we detected increased migration when measured with the Boyden-chamber based Roche xCelligence assay (Fig. 5B). During passaging, we observed that IH-selected MCF10A clones detached easily by treatment with trypsin, while unselected or parental cultures required long incubations in trypsin to detach. While we do not detect changes in morphology amongst individual cells, we detected a clear mesynchemal phenotype in intermittent hypoxic selected cells (Fig. 5C) compared to controls. Despite these data that suggest transformation, however, MCF10A cells injected into the mammary fat pads of nude mice did not develop into tumors (S10 Fig.). This may be due to decreased proliferation of IH-selected cells (Fig. 5D).

To measure metabolic changes following IH-selection, we employed the XF-96 Seahorse Analysis system. This instrument measures oxygen consumption and glucose-stimulated proton production in real-time *in vitro*. We had previously hypothesized that, as an adaptation to hypoxia, that IH-selected cells would exhibit increased aerobic glycolysis (Warburg Effect) [45]. However, contrary to our hypothesis, IH-selected clones exhibited an increased amount of energy generation from respiration compared to control cells (Fig. 5E). In a study published in 2010, the Vogelstein group reported that selection by glucose deprivation led outgrowth of KRAS mutated pancreatic cells that had an associated increase in glycolysis [46]. In an attempt to recapitulate their finding in our system, we subjected MCF10A cells to 0.5mM Glucose and hypoxia in acidic media for 2 weeks. While this treatment was toxic to the vast majority of the cells, upon re-growth, we detected that several clones had increased glycolysis over control cells (S11 Fig.).

## Discussion

In this report, we simulated the microenvironment that exists in early carcinogenesis to determine the long-term effects of these conditions on the emergence of stable phenotypes in multiple cell types. Significantly, we demonstrated the acquisition of novel phenotypes and associated genotypes through natural mechanisms that mimic those *in vivo*. This work serves as a model of carcinogenesis that may describe the evolutionary dynamics of cells as they acquire malignant phenotypes. An understanding of the mechanisms of carcinogenesis is crucial to the development of novel preventive therapies.

The process of carcinogenesis is speculated to occur during a decades-long period [47, 48]. Notably, microenvironmental perturbations due to chronic inflammation can drive initiation of carcinogenesis [49]. Chronic inflammation induces hypoxia and acidosis, and adaptation to these conditions potentially leads to changes in survivability and proliferation. Following initiation and hyperplasia, hypoxia is present during the *in situ* carcinoma stage due to diffusion limitations as cells proliferate into the lumen away from the underlying tissue. Further, as cancers grow and locally invade, they experience regional hypoxia that is also known to be periodic. Thus, in this study, we attempted to recapitulate those conditions and test their effects on cells that represent different stages of cancer. Notably, we observed the largest changes in pre-malignant cells indicating that they may have the greatest adaptive leap to make.

Previous studies have demonstrated clonal outgrowths of minor populations with growth advantages. These studies are important in understanding evolutionary progression of cancers and how certain genotypes are expressed. For example, p53 null cells, even when cultured 1:1000 relative to p53 wild-type cells, can outgrow the p53 wild-type cells in as selective environment, such as hypoxia [11]. This type of study helps us understand how when a single cell acquires a beneficial mutation it can eventually dominate the population and that this behavior is contextual i.e. it requires a selective microenvironment. Additionally, scores of other studies have demonstrated that random mutagenesis with genotoxic agents can lead to the acquisition of new genotypes. Our study connects these two observations using a mutagenesis process that models naturally occurring conditions.

Hypoxia and intermittent hypoxia are now commonly recognized as two physiologically distinct processes [16, 50]. While chronic (diffusion-limited) hypoxia is a result of distance from the vasculature and is common among large tumors, intermittent (perfusion-limited) hypoxia is a result from fluctuating vasculature. These dissimilarities lead to differences in intracellular signaling, therapeutic resistance, and progression. Molecularly, this is manifested in differing roles of HIF-1[50] In the current study, we sought to define adaptation to intermittent hypoxia, as this has been reported to produce more severe phenotypes. While we observed vast changes in MCF10A cells following treatment with intermittent hypoxia, we observed few changes in chronically (60 day) treated cells. We hypothesize that this may be due to the fact that chronic hypoxia is not as mutagenic or as selective as intermittent hypoxia.

We observed the acquisition of constitutive CA-IX expression in cells following intermittent hypoxia. Constitutive CA-IX expression is commonly observed in cancers but not in normal cells in which CA-IX is expressed only during hypoxia. The molecular mechanism through which this occurs is still unclear. Nevertheless, it is clear that CA-IX expression is associated with a poor prognosis [51–54]. Additionally, CA-IX expression does not always correlate with hypoxia *in vivo*, a condition known as “pseudohypoxia”, wherein hypoxia activated gene products are expressed under normoxic conditions Our work suggests that intermittent hypoxia leads to constitutive CA-IX expression, suggesting that tumor cells in normoxic regions may have once resided within intermittent hypoxia regions of the tumor.

It is also important to note that different tumors exhibit varying adaptive landscapes. For example, not all tumors are hypoxic, therefore not all cancerous cells will adapt to hypoxia as the cells adapted in the present study. Notably, tumors of SU86.86 cells are highly oxygenated. Hence, adaptations to hypoxia, such as loss of E-cadherin, would not have occurred during the evolution of this cancer. While hypoxia may not be necessary for loss of E-cadherin, this study indicates that it serves as a strong selective pressure for its loss. It remains a mystery why hypoxia would select for loss of E-cadherin. One possible explanation is that loss of E-cadherin promotes invasion, thus allowing cells to move to regions that are more well-oxygenated. While this may be obvious *in vivo*, it is less so *in vitro*. However, it is also known that tissue culture plastic can sequester O_2_, and hence a more migratory phenotype may be able to interrogate a greater region of oxygen-containing space. The loss of p53 is more obvious, as hypoxia is a potent selector for apoptosis deficiencies.

The detection of EMT in MCF10A IH-selected clones led us to hypothesize that these clones would grow tumors *in vivo*. This turned out to not be the case. We must conclude that the IH-selected cells have not acquired the sufficient number of oncogenic events to form tumors and are thus intermediate in oncogenic progression between MCF10A cells and MCF10.DCIS cells. Another observation may be relevant to tumorigenicity is the increase in respiratory capacity of IH-selected cells. Generally, as cells progress to carcinogenicity, they acquire a glycolytic phenotype. We have hypothesized that aerobic glycolysis results from intermittent hypoxia [45]. From the results of this study, we conclude that intermittent hypoxia itself is not sufficient for the acquisition of the glycolytic phenotype. The increased respiration of IH-selected cells is an artifact of the system that may be due to high glucose that is not present *in vivo*. In order for the glycolytic phenotype to be obtained, reduced glucose must be present, an observation previously reported by Vogelstein’s group [46].The microenvironmental conditions present within a tumor can be used as prognostic factors [54–56]. This study suggests that if a set of microenvironmental conditions leads to invasion or to drug resistance, then detection of those in a tumor could lead to better risk assessment for local invasion. In future experiments, we intend to test additional microenvironmental conditions in directing evolution. We hope that approaches to perturb the microenvironment, as through buffer therapy, to direct the evolution of the tumor can be realized [57–59].

## Methods

### Animal care and use

All procedures were approved by the Institutional Animal Care and Use Committee of University of South Florida under the approved protocol R4029, and animals were maintained in accordance with IACUC standards of care in pathogen free rooms, in the USF Vivarium on site at the Moffitt Cancer Research Center. Female athymic nude mice were acquired from Harlan Laboratories. Four mice were injected in the right (R) flank with MCF10A-IH clone 4, and on the left (F) flank with non selected MCF10A clone 4. The other four mice were injected on the R flank with MCF10A IH clone 9 and on the L flank with MCF10A control clone 7. Mice were imaged at the time of injection and weekly thereafter for 4 weeks.

### Cell lines and Culture Conditions

MCF10.DCIS, and MDA-MB-231 were obtained through the Physical Sciences Oncology Center at the NIH (PSOC, National Institutes of Health, Bethesda, MD). MCF10A, SU86.86, and RKO cells were obtained through American Type Culture Collection (ATCC). MCF10A cells were grown in DMEM/F12 supplemented with 5% Horse Serum, 0.5μg/mL hydrocortisone, 100ng/mL cholera toxin, 20ng/mL human epidermal growth factor, and 10μg/mL insulin. MCF10.DCIS cells were grown in DMEM/F12 supplemented with 5% Horse Serum. MDA-MB-231 cells were grown in DMEM/F12 supplemented with 10% FBS. SU86.86 cells were grown in RPMI supplemented with 10% FBS. RKO Cells were grown in DMEM supplemented with 10% FBS. All cells were grown in 5% CO_2_ at all times. These growth conditions for each cell line were used during normal growth, hypoxic treatment, and during clonal selection. Stable transfection of MCF10A with plasmid containing firefly luciferase was maintained with constant culture of 10ug/mL Blasticidin Testing for mycoplasma was performed using the Lonza MycoAlert Mycoplasma Detection Kit (Cat # LT07–218).

### Intermittent hypoxia and chronic hypoxia selection

The Biospherix X-Vivo Hypoxia Chamber was used for all experiments. During hypoxic treatments, cells were incubated at 37°C, 5% CO_2_, with either 0.2% O_2_ and 94.8% N_2_ or 1.0% O_2_ and 95% N_2_. Reoxygenation was performed by transferring flasks or plates containing cells from the hypoxic chamber to an incubator under atmospheric conditions at 5%CO_2_. Chronic hypoxia treated cells were cultured constantly at 0.2% O_2._ The chamber was quality controlled and calibrated according to the manufacturer’s specifications (Biospherix). Additional validation of oxygen tension was performed using the CHEMets Dissolved Oxygen Kit (CHEMetrics).

### Development of clonal populations

Individual cells were diluted to single-cells suspensions and grown in each well in 96-well plates under normal growth conditions. Any well containing the growth of more than one colony was excluded. Upon confluency, colonies were isolated and grown under normal growth conditions.

### Real Time RT-PCR

For all real-time PCR reactions, the iScript Real-Rime PCR kit with SYBR Green (Cat# 170–8893 Biorad) was used on a HT Fast-Real-Time System (Applied Biosciences). Primers were derived from Primer Depot (primerdepot.nci.nih.gov/). Annealing temperatures used were based on the computationally predicted annealing temperature provided by Primer Depot. 100ng of mRNA was used per 20uL reaction. Trizol (Invitrogen) was used according to the manufacturer’s directions for all RNA purification. Amplification of GAPDH or actin was used as an internal control for all experiments.

### Western Blotting

Cell pellets were lysed in RIPA buffer and electrophoresed on 8 or 10% polyacrylamide gels. Primary antibodies used for western blotting were: for p53 Cat# OP43 (Oncogene), for actin Cat# SC-1616 (Santa-Cruz Biotechnology), for N-cadherin #G10920 (BD Biosciences), and for E-cadherin #610181 (BD Biosciences), for HIF-1α Cat#SC-10790 (Santa-Cruz Biotechnology), for GLUT1 Cat# SC-7903 (Santa-Cruz Biotechnology), for CA-IX Cat# PA-513086 (Thermo Scientific), and for GAPDH Cat# PA1–988 (Thermo Scientific).

### Karyotyping

Nanostring nCounter karyotyping was performed according to the manufacturer’s specifications (Nanostring, Seattle WA).

### Cytotoxic Assays

In all reported assays, cells were exposed to cytotoxins for 72hrs. Cells were treated with 10nM docetaxel, 20μM etoposide, or 10nM methotrexate or were exposed to pH 6.7 or 0.2% O_2_ and then viability was measured by the crystal violet assay and compared to untreated cultures. For MCF10A IH-selected cells, growth-factor independence was tested by culturing in media lacking hEGF.

### Metabolic analysis

We measured oxygen consumption and proton production glycolysis using the 96-well XF Seahorse Analysis system (Seahorse Biosciences, Massachusetts). To measure maximum oxygen consumption, cells were treated with 2.5 μM FCCP. For glucose consumption, cells were treated with rotenone and antimycin to measure maximum glycolysis following basal readings.

### Immunohistochemical staining, imaging and analysis

Slides were stained using a Ventana Discovery XT automated system (Ventana Medical Systems, Tucson) as per manufacturer's protocol with proprietary reagents. To stain for CAIX, the rabbit primary antibody #ab15086 (Abcam, Cambridge, MA) was used at a 1:500 concentration in Dako antibody diluent and incubated for 32 minutes. The same concentration and times were used for GLUT-1 ab#652 and HIF-1α ab#1. The OmniMap anti-rabbit secondary was used for 20 minutes. ChromoMap was used for detection.

Immunostained slides of DCIS lesions stained against GLUT-1, HIF1α and CA-IX were scanned using the Aperio (Vista, CA) ScanScope XT with a 200x/0.8NA objective lens at a rate of 5 minutes per slide via Basler trilinear array. TissueStudio (Definiens, Munich, Germany) image analysis platform was used to segment, classify and extract size and intensity features for single cells in the viable tumor regions and classified into negative, weak, moderate and strong intensity. Each biomarker analysis was specific to its orientation within the cells (GLUT1 and HIF1α on the membrane; CA-IX in the cytoplasm) and quality controlled by a practicing pathologist.

### Reverse Phase Protein Array (RPPA)

Protein expression for a total of eight MCF10A cells (2 IH-selected and 2 normal, each done in duplicate) was obtained through The MD Anderson Cancer Center. The assay evaluated 214 protein markers and the assay had positive and negative controls. Sample preparation was performed according to the facility’s recommendations. Briefly, samples were lysed in RIPA buffer containing Phosphatase inhibitors and protease inhibitors and frozen. For data analysis, the assay was normalized (linear methods based on internal controls), median centered and log2 transformed prior to analysis. Students T-test (Welch) was performed to find significant markers between Normal and Hypoxic groups. In order to control the familywise multiple testing errors, the False discovery rate (FDR) was computed [40, 41].

## Supporting Information

S1 Fig GLUT-1 is present at the invasive front. Immunohistochemical staining of GLUT-1 of an invasive breast tumor. GLUT-1 (brown) is highly expressed peri-luminally, and along the invasive front.
(TIF)

S2 Fig Cell survival in chronic and intermittent hypoxia. Sensitivity to chronic and intermittent hypoxia of (A) metastatic breast cancer MDA-MB—231, (B) breast epithelial MCF10.DCIS, (C) metastatic pancreatic SU86.86, and (D) Colorectal RKO cells. Cells were incubated in normoxia, chronic hypoxia, or intermittent for 6 days, and relative survival was measured to cells grown in normoxia.
(TIF)

S3 Fig IH modulates MDA-MB-231 survival to etoposide. Sensitivity of IH-selected and passage control MCF10.DCIS and MDA-MB-231 clones. While IH-selection did not decrease etoposide resistance for MCF10.DCIS cells (A), MDA-MB-231 clones exhibited a significant increase in resistance (B).
(TIF)

S4 Fig IH leads to changes in p53 and e-cadherin expression following 2 weeks of selection. MCF10A cells were seeded at 10kcells/well in 8 wells of 12-well plates and treated with either reduced glucose and pH, or IH daily, or grown in normal growth conditions for 2 weeks, and mRNA was isolated from each clone. (A) While treatment with a control condition (0.5mM Glucose, pH 6.8) did not reduce expression of p53, treatment with IH reduced expression of p53. (B) Treatment with IH also significantly reduced the expression of E-cadherin.
(TIF)

S5 Fig Selection by chronic hypoxia, unlike IH, does not lead to changes in e-cadherin or p53. No significant differences in E-cadherin or p53 expression were detected following treatment with chronic hypoxia. RNA was isolated from 10 Chronic Hypoxia treated clones and 10 passaged control clones. mRNA levels of p53, E-cadherin, and actin was measured by quantitative RT-PCR.
(TIF)

S6 Fig No significant changes in survival were detected in chronic hypoxia adapted cells when compared to control passaged cells. Chronic hypoxia treated clones and control clones were treated with 20μM etoposide, 10nM Docetaxel, 0.2% O_2_, or untreated in normal growth conditions for 72 hours. No significant difference in survival under any of these conditions was measured.
(TIF)

S7 Fig Expression of GAPDH is not significantly affected by intermittent hypoxia selection. mRNA was isolated from MCF10A parental control cells, MCF10A parental IH-selected cells, and MCF10a IH selected clone 4 and MCF10A IH-selected clone 9. Fold expression of GAPDH over actin as a loading control was measured. No significant difference was measured for MCF10A IH-selected parental cells (p = 0.9400) or IH-selected clones 4 and 9 (p = .9751).
(TIF)

S8 Fig Expression of CA-IX in MDA-MB-231, MCF10.DCIS, or MCF10A cells while in culture in normoxia, IH, or hypoxia. All cells were cultured for 6 days, then RNA was extracted, followed by semi-quantitative PCR to measure CA-IX expression. MDA-MB-231 cells exhibit some constitutive CA-IX expression that is increased under IH and hypoxia. DCIS cells constitutively express about the same amount of CA-IX. MCF10A cells only express CA-IX while under IH or hypoxia.
(TIF)

S9 Fig qRT-PCR and semi-quantitative PCR of mRNA from IH-selected clones 4 and 9, and control clones 4 an7. IH-selected clones exhibited an increase in CA-IX expression. Detection of CA-IX by western blot revealed a modest increase following IH-selection.
(TIF)

S10 Fig IH is not sufficient for tumorigenesis *in vivo*. MCF10A-luc control and IH selected cells were injected subcutaneously in to the right and left flank of Nu/Nu male mouse (n = 4) at a concentration of 1–5x10^7^. Mean tumor bioluminescence was quantified in each group indicating no growth difference between the two cohorts at 4 weeks post injections.
(TIF)

S11 Fig IH in combination with reduced glucose and reduced pH may lead to increased glycolysis. MCF10A cells were subjected to either standard growth conditions (25mM glucose, pH 7.2, 21%O_2_) or to low glucose, low pH, hypoxic conditions (0.5mM glucose, pH 6.7, 0.2% O_2_) for two weeks. Metabolic analysis of clones of the low pH low glucose, hypoxic growth cells (teal) exhibited increased glycolysis relative to control cells (blue).
(TIF)

## References

[1] Hanahan D, Weinberg RA. Hallmarks of cancer: the next generation. Cell. 2011;144(5):646–674. 10.1016/j.cell.2011.02.013 21376230

[2] Gatenby RA, Gillies RJ. A microenvironmental model of carcinogenesis. Nature reviews. 2008;8(1):56–61. 18059462 10.1038/nrc2255

[3] Fletcher GH. Radiation and drug resistance of breast cancer. Am J Clin Oncol. 1984;7(6):617–624. 6528860 10.1097/00000421-198412000-00007

[4] Gonzalez-Angulo AM, Morales-Vasquez F, Hortobagyi GN. Overview of resistance to systemic therapy in patients with breast cancer. Adv Exp Med Biol. 2007;608:1–22. 17993229 10.1007/978-0-387-74039-3_1

[5] Zhou J, Schmid T, Schnitzer S, Brune B. Tumor hypoxia and cancer progression. Cancer Lett. 2006;237(1):10–21. 16002209 10.1016/j.canlet.2005.05.028

[6] Hockel M, Vaupel P. Biological consequences of tumor hypoxia. Semin Oncol. 2001;28(2 Suppl 8):36–41. 11395851

[7] Aktipis CA, Kwan VS, Johnson KA, Neuberg SL, Maley CC. Overlooking evolution: a systematic analysis of cancer relapse and therapeutic resistance research. PLOS One. 2011;6(11):e26100 10.1371/journal.pone.0026100 22125594 PMC3219640

[8] Wojtkowiak JW, Verduzco D, Schramm KJ, Gillies RJ. Drug resistance and cellular adaptation to tumor acidic pH microenvironment. Mol Pharm. 2011;8(6):2032–2038. 10.1021/mp200292c 21981633 PMC3230683

[9] Cardaci S, Ciriolo MR. TCA Cycle Defects and Cancer: When Metabolism Tunes Redox State. International journal of cell biology. 2012;2012:161837 10.1155/2012/161837 22888353 PMC3408673

[10] Cattaneo MG, Cappellini E, Benfante R, Ragni M, Omodeo-Sale F, Nisoli E, et al Chronic deficiency of nitric oxide affects hypoxia inducible factor-1alpha (HIF-1alpha) stability and migration in human endothelial cells. PLOS One. 2011;6(12):e29680 10.1371/journal.pone.0029680 22216344 PMC3246476

[11] Graeber TG, Osmanian C, Jacks T, Housman DE, Koch CJ, Lowe SW, et al Hypoxia-mediated selection of cells with diminished apoptotic potential in solid tumours. Nature. 1996;379(6560):88–91. 8538748 10.1038/379088a0

[12] Bindra RS, Glazer PM. Genetic instability and the tumor microenvironment: towards the concept of microenvironment-induced mutagenesis. Mutat Res. 2005;569(1–2):75–85. 15603753 10.1016/j.mrfmmm.2004.03.013

[13] Gerlinger M, Rowan AJ, Horswell S, Larkin J, Endesfelder D, Gronroos E, et al Intratumor heterogeneity and branched evolution revealed by multiregion sequencing. N Engl J Med. 2012;366(10):883–892. 10.1056/NEJMoa1113205 22397650 PMC4878653

[14] Yachida S, Jones S, Bozic I, Antal T, Leary R, Fu B, et al Distant metastasis occurs late during the genetic evolution of pancreatic cancer. Nature. 2010;467(7319):1114–1117. 10.1038/nature09515 20981102 PMC3148940

[15] Sottoriva A, Spiteri I, Piccirillo SG, Touloumis A, Collins VP, Marioni JC, et al Intratumor heterogeneity in human glioblastoma reflects cancer evolutionary dynamics. Proc Natl Acad Sci U S A. 2013;110(10):4009–4014. 10.1073/pnas.1219747110 23412337 PMC3593922

[16] Bayer C, Vaupel P. Acute versus chronic hypoxia in tumors: Controversial data concerning time frames and biological consequences. Strahlenther Onkol. 2012;188(7):616–627. 10.1007/s00066-012-0085-4 22454045

[17] Wykoff CC, Beasley NJ, Watson PH, Turner KJ, Pastorek J, Sibtain A, et al Hypoxia-inducible expression of tumor-associated carbonic anhydrases. Cancer Res. 2000;60(24):7075–7083. 11156414

[18] Airley R, Loncaster J, Davidson S, Bromley M, Roberts S, Patterson A, et al Glucose transporter glut-1 expression correlates with tumor hypoxia and predicts metastasis-free survival in advanced carcinoma of the cervix. Clin Cancer Res. 2001;7(4):928–934. 11309343

[19] Helmlinger G, Yuan F, Dellian M, Jain RK. Interstitial pH and pO2 gradients in solid tumors in vivo: high-resolution measurements reveal a lack of correlation. Nature medicine. 1997;3(2):177–182. 9018236 10.1038/nm0297-177

[20] Dewhirst MW, Kimura H, Rehmus SW, Braun RD, Papahadjopoulos D, Hong K, et al Microvascular studies on the origins of perfusion-limited hypoxia. Br J Cancer Suppl. 1996;27(51):S247–251. 8763890 PMC2149984

[21] Braun RD, Lanzen JL, Dewhirst MW. Fourier analysis of fluctuations of oxygen tension and blood flow in R3230Ac tumors and muscle in rats. Am J Physiol. 1999;277(2 Pt 2):H551–568. 10444480 10.1152/ajpheart.1999.277.2.H551

[22] Baudelet C, Cron GO, Ansiaux R, Crokart N, DeWever J, Feron O, et al The role of vessel maturation and vessel functionality in spontaneous fluctuations of T2*-weighted GRE signal within tumors. NMR in biomedicine. 2006;19(1):69–76. 16411170 10.1002/nbm.1002

[23] Gilead A, Neeman M. Dynamic remodeling of the vascular bed precedes tumor growth: MLS ovarian carcinoma spheroids implanted in nude mice. Neoplasia. 1999;1(3):226–230. 10935477 10.1038/sj.neo.7900032 PMC1508074

[24] Bindra RS, Crosby ME, Glazer PM. Regulation of DNA repair in hypoxic cancer cells. Cancer Metastasis Rev. 2007;26(2):249–260. 17415527 10.1007/s10555-007-9061-3

[25] Meng AX, Jalali F, Cuddihy A, Chan N, Bindra RS, Glazer PM, et al Hypoxia down-regulates DNA double strand break repair gene expression in prostate cancer cells. Radiother Oncol. 2005;76(2):168–176. 16026872 10.1016/j.radonc.2005.06.025

[26] Young SD, Marshall RS, Hill RP. Hypoxia induces DNA overreplication and enhances metastatic potential of murine tumor cells. Proc Natl Acad Sci U S A. 1988;85(24):9533–9537. 3200838 10.1073/pnas.85.24.9533 PMC282788

[27] Greijer AE, van der Wall E. The role of hypoxia inducible factor 1 (HIF-1) in hypoxia induced apoptosis. J Clin Pathol. 2004;57(10):1009–1014. 15452150 10.1136/jcp.2003.015032 PMC1770458

[28] Santore MT, McClintock DS, Lee VY, Budinger GR, Chandel NS. Anoxia-induced apoptosis occurs through a mitochondria-dependent pathway in lung epithelial cells. Am J Physiol Lung Cell Mol Physiol. 2002;282(4):L727–734. 11880298 10.1152/ajplung.00281.2001

[29] Gillies RJ, Verduzco D, Gatenby RA. Evolutionary dynamics of carcinogenesis and why targeted therapy does not work. Nature reviews. 2012;12(7):487–493. 10.1038/nrc3298 22695393 PMC4122506

[30] Pires IM, Bencokova Z, Milani M, Folkes LK, Li JL, Stratford MR, et al Effects of acute versus chronic hypoxia on DNA damage responses and genomic instability. Cancer Res. 2010;70(3):925–935. 10.1158/0008-5472.CAN-09-2715 20103649 PMC2923514

[31] Piret JP, Cosse JP, Ninane N, Raes M, Michiels C. Hypoxia protects HepG2 cells against etoposide-induced apoptosis via a HIF-1-independent pathway. Exp Cell Res. 2006;312(15):2908–2920. 16844113 10.1016/j.yexcr.2006.05.018

[32] Cosse JP, Sermeus A, Vannuvel K, Ninane N, Raes M, Michiels C. Differential effects of hypoxia on etoposide-induced apoptosis according to the cancer cell lines. Mol Cancer. 2007;6:61 17894897 10.1186/1476-4598-6-61 PMC2099441

[33] Rice GC, Hoy C, Schimke RT. Transient hypoxia enhances the frequency of dihydrofolate reductase gene amplification in Chinese hamster ovary cells. Proc Natl Acad Sci U S A. 1986;83(16):5978–5982. 3461470 10.1073/pnas.83.16.5978 PMC386420

[34] Hanahan D, Weinberg RA. The hallmarks of cancer. Cell. 2000;100(1):57–70. 10647931 10.1016/s0092-8674(00)81683-9

[35] Nollet F, Berx G, van Roy F. The role of the E-cadherin/catenin adhesion complex in the development and progression of cancer. Mol Cell Biol Res Commun. 1999;2(2):77–85. 10542129 10.1006/mcbr.1999.0155

[36] Kadota M, Yang HH, Gomez B, Sato M, Clifford RJ, Meerzaman D, et al Delineating genetic alterations for tumor progression in the MCF10A series of breast cancer cell lines. PLOS One. 2010;5(2):e9201 10.1371/journal.pone.0009201 20169162 PMC2821407

[37] Wojtkowiak JW, Rothberg JM, Kumar V, Schramm KJ, Haller E, Proemsey JB, et al Chronic autophagy is a cellular adaptation to tumor acidic pH microenvironments. Cancer Res. 2012;72(16):3938–3947. 10.1158/0008-5472.CAN-11-3881 22719070 PMC3749826

[38] Brazdova M, Navratilova L, Tichy V, Nemcova K, Lexa M, Hrstka R, et al Preferential binding of hot spot mutant p53 proteins to supercoiled DNA in vitro and in cells. PLOS One. 2013;8(3):e59567 10.1371/journal.pone.0059567 23555710 PMC3608670

[39] Vikhanskaya F, Siddique MM, Kei Lee M, Broggini M, Sabapathy K. Evaluation of the combined effect of p53 codon 72 polymorphism and hotspot mutations in response to anticancer drugs. Clin Cancer Res. 2005;11(12):4348–4356. 15958617 10.1158/1078-0432.CCR-04-1547

[40] Benjamini Y, Hochberg Y. Controlling the false discovery rate: A practical and powerful approach to multiple testing. J R Stat Soc Series B Stat Methadol. 1995;57(1):289–300.

[41] Storey JD, Tibshirani R. Statistical significance for genomewide studies. Proc Natl Acad Sci U S A. 2003;100(16):9440–9445. 12883005 10.1073/pnas.1530509100 PMC170937

[42] Tsai YP, Wu KJ. Hypoxia-regulated target genes implicated in tumor metastasis. J Biomed Sci. 2012;19:102 10.1186/1423-0127-19-102 23241400 PMC3541338

[43] Yang MH, Wu MZ, Chiou SH, Chen PM, Chang SY, Liu CJ, et al Direct regulation of TWIST by HIF-1alpha promotes metastasis. Nat Cell Biol. 2008;10(3):295–305. 10.1038/ncb1691 18297062

[44] Ginzinger DG, Godfrey TE, Nigro J, Moore DH 2nd, Suzuki S, Pallavicini MG, et al Measurement of DNA copy number at microsatellite loci using quantitative PCR analysis. Cancer Res. 2000;60(19):5405–5409. 11034080

[45] Gatenby RA, Gillies RJ. Why do cancers have high aerobic glycolysis? Nature reviews. 2004;4(11):891–899. 15516961 10.1038/nrc1478

[46] Yun J, Rago C, Cheong I, Pagliarini R, Angenendt P, Rajagopalan H, et al Glucose deprivation contributes to the development of KRAS pathway mutations in tumor cells. Science. 2009;325(5947):1555–1559. 10.1126/science.1174229 19661383 PMC2820374

[47] Yachida S, Iacobuzio-Donahue CA. Evolution and dynamics of pancreatic cancer progression. Oncogene. 2013;18(10):29. 10.1038/onc.2013.29 PMC3823715 23416985

[48] Lengauer C, Kinzler KW, Vogelstein B. Genetic instabilities in human cancers. Nature. 1998;396(6712):643–649. 9872311 10.1038/25292

[49] Gillies RJ, Robey I, Gatenby RA. Causes and consequences of increased glucose metabolism of cancers. J Nucl Med. 2008;49 Suppl 2(2):24S–42S. 10.2967/jnumed.107.047258 18523064

[50] Dewhirst MW. Intermittent hypoxia furthers the rationale for hypoxia-inducible factor-1 targeting. Cancer Res. 2007;67(3):854–855. 17283112 10.1158/0008-5472.CAN-06-4744

[51] Giatromanolaki A, Koukourakis MI, Sivridis E, Pastorek J, Wykoff CC, Gatter KC, et al Expression of hypoxia-inducible carbonic anhydrase-9 relates to angiogenic pathways and independently to poor outcome in non-small cell lung cancer. Cancer Res. 2001;61(21):7992–7998. 11691824

[52] Chia SK, Wykoff CC, Watson PH, Han C, Leek RD, Pastorek J, et al Prognostic significance of a novel hypoxia-regulated marker, carbonic anhydrase IX, in invasive breast carcinoma. J Clin Oncol. 2001;19(16):3660–3668. 11504747 10.1200/JCO.2001.19.16.3660

[53] Generali D, Berruti A, Brizzi MP, Campo L, Bonardi S, Wigfield S, et al Hypoxia-inducible factor-1alpha expression predicts a poor response to primary chemoendocrine therapy and disease-free survival in primary human breast cancer. Clin Cancer Res. 2006;12(15):4562–4568. 16899602 10.1158/1078-0432.CCR-05-2690

[54] Jubb AM, Buffa FM, Harris AL. Assessment of tumour hypoxia for prediction of response to therapy and cancer prognosis. J Cell Mol Med. 2010;14(1–2):18–29. 19840191 10.1111/j.1582-4934.2009.00944.x PMC3837600

[55] Gillies RJ, Gatenby RA. Hypoxia and adaptive landscapes in the evolution of carcinogenesis. Cancer Metastasis Rev. 2007;26(2):311–317. 17404691 10.1007/s10555-007-9065-z

[56] Gatenby RA, Smallbone K, Maini PK, Rose F, Averill J, Nagle RB, et al Cellular adaptations to hypoxia and acidosis during somatic evolution of breast cancer. Br J Cancer. 2007;97(5):646–653. 17687336 10.1038/sj.bjc.6603922 PMC2360372

[57] Ibrahim-Hashim A, Cornnell HH, Abrahams D, Lloyd M, Bui M, Gillies RJ, et al Systemic buffers inhibit carcinogenesis in TRAMP mice. J Urol. 2012;188(2):624–631. 10.1016/j.juro.2012.03.113 22704445 PMC3694604

[58] Bailey KM, Wojtkowiak JW, Hashim AI, Gillies RJ. Targeting the metabolic microenvironment of tumors. Adv Pharmacol. 2012;65:63–107. 10.1016/B978-0-12-397927-8.00004-X 22959024 PMC3796340

[59] Gatenby RA, Gillies RJ, Brown JS. Evolutionary dynamics of cancer prevention. Nature reviews. 2010;10(8):526–527. 21137109 10.1038/nrc2892 PMC3744108

