# Supplementary figures and images for: Intermittent Hypoxia Selects for Genotypes and Phenotypes That Increase Survival, Invasion, and Therapy Resistance
Source: PLoS One. 2015 Mar 26;10(3):e0120958. doi: 10.1371/journal.pone.0120958 (PMC4374837; doi:10.1371/journal.pone.0120958)

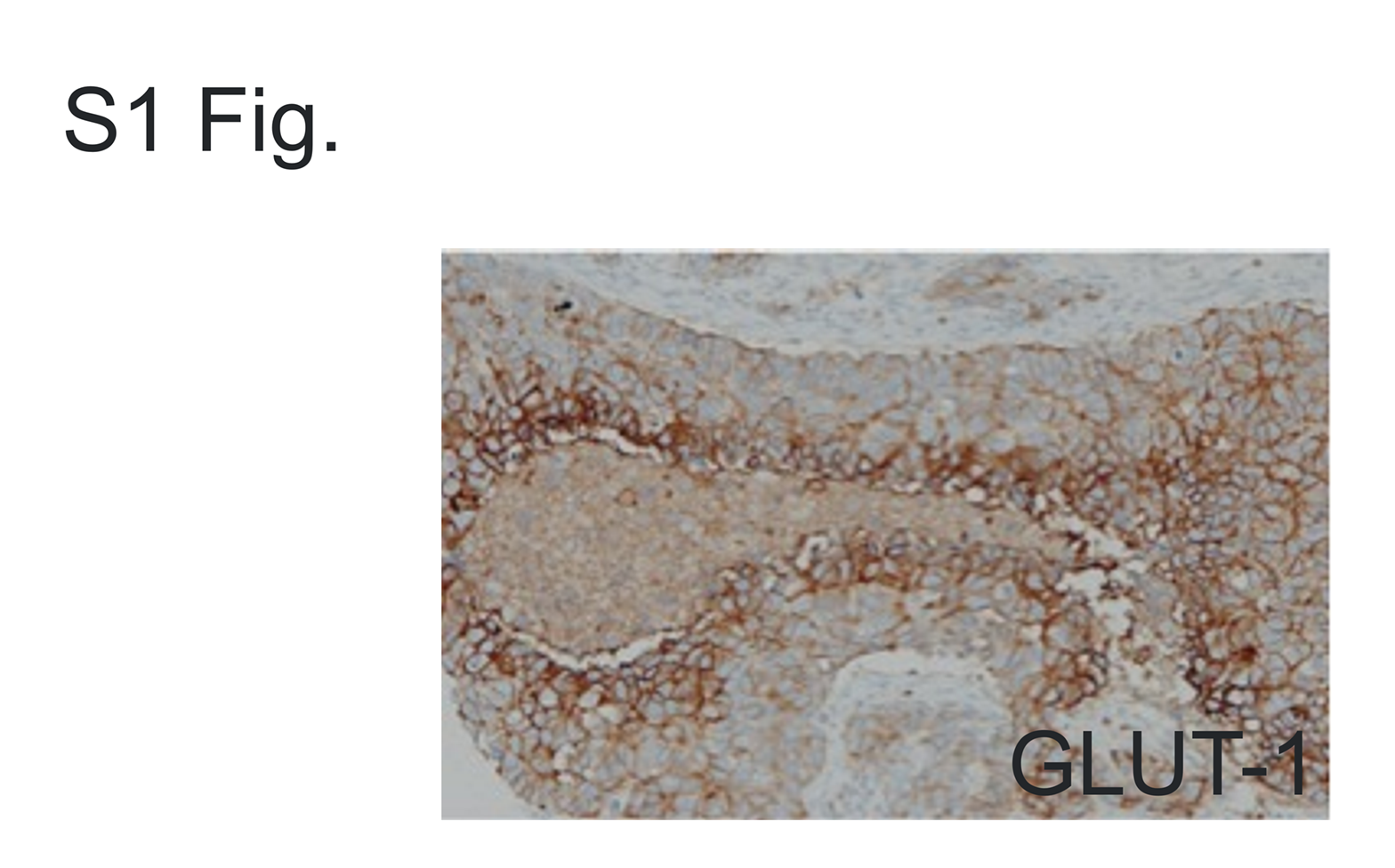

Supplement: S1 Fig — Immunohistochemical staining of GLUT-1 of an invasive breast tumor. GLUT-1 (brown) is highly expressed peri-luminally, and along the invasive front. (TIF) [file pone.0120958.s001.tif]

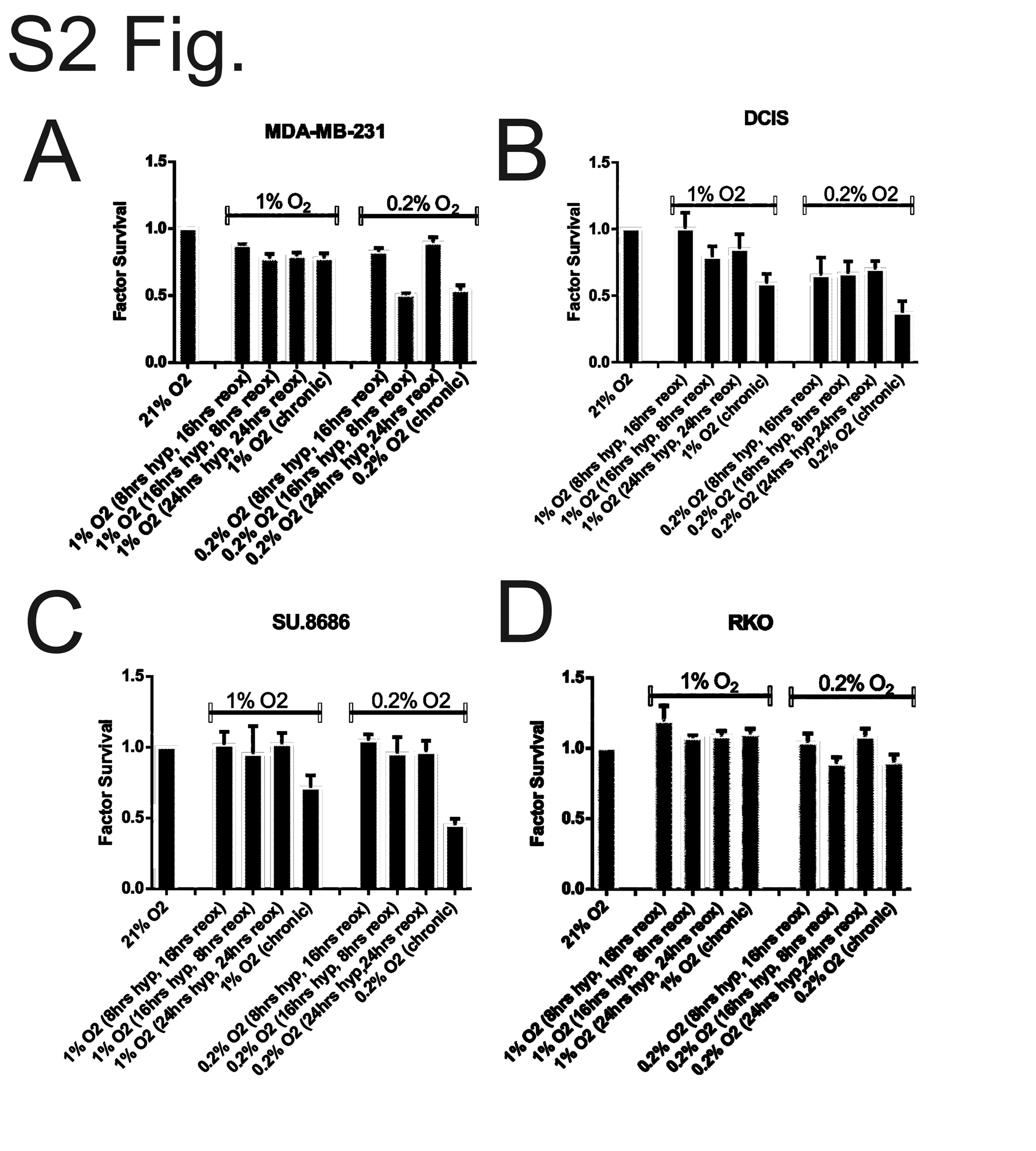

Supplement: S2 Fig — Sensitivity to chronic and intermittent hypoxia of (A) metastatic breast cancer MDA-MB—231, (B) breast epithelial MCF10.DCIS, (C) metastatic pancreatic SU86.86, and (D) Colorectal RKO cells. Cells were incubated in normoxia, chronic hypoxia, or intermittent for 6 days, and relative survival was measured to cells grown in normoxia. (TIF) [file pone.0120958.s002.tif]

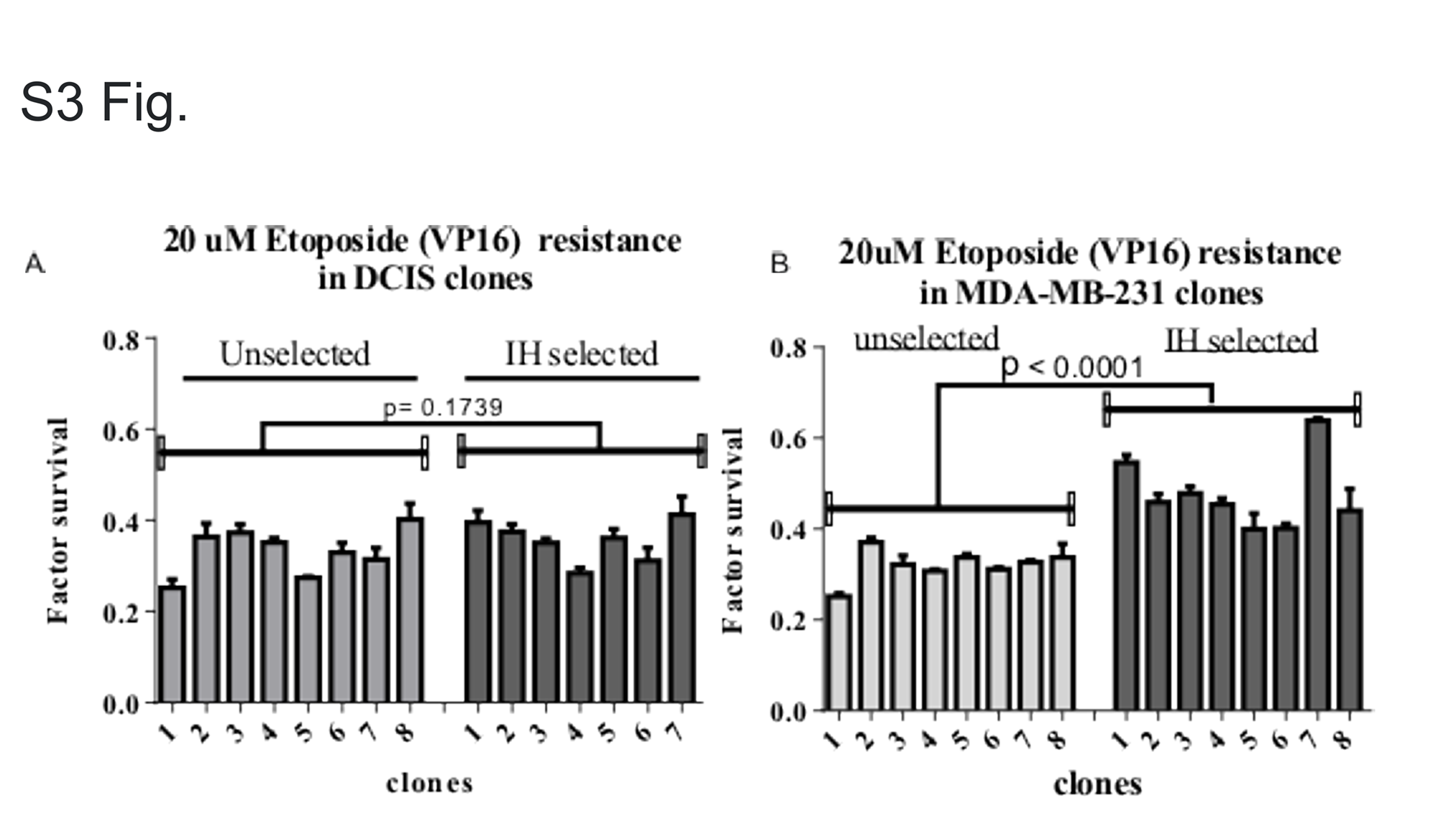

Supplement: S3 Fig — Sensitivity of IH-selected and passage control MCF10.DCIS and MDA-MB-231 clones. While IH-selection did not decrease etoposide resistance for MCF10.DCIS cells (A), MDA-MB-231 clones exhibited a significant increase in resistance (B). (TIF) [file pone.0120958.s003.tif]

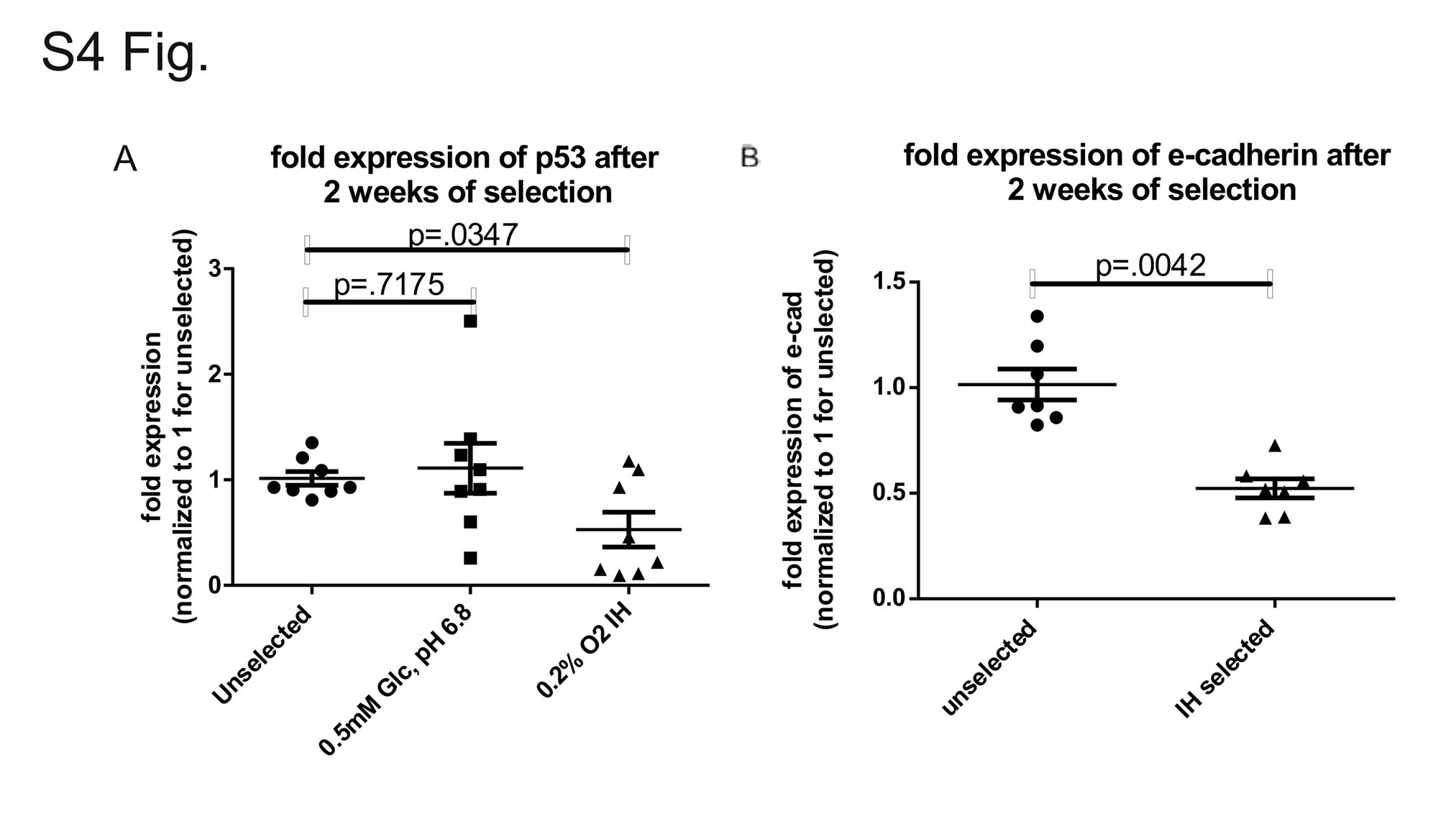

Supplement: S4 Fig — MCF10A cells were seeded at 10kcells/well in 8 wells of 12-well plates and treated with either reduced glucose and pH, or IH daily, or grown in normal growth conditions for 2 weeks, and mRNA was isolated from each clone. (A) While treatment with a control condition (0.5mM Glucose, pH 6.8) did not reduce expression of p53, treatment with IH reduced expression of p53. (B) Treatment with IH also significantly reduced the expression of E-cadherin. (TIF) [file pone.0120958.s004.tif]

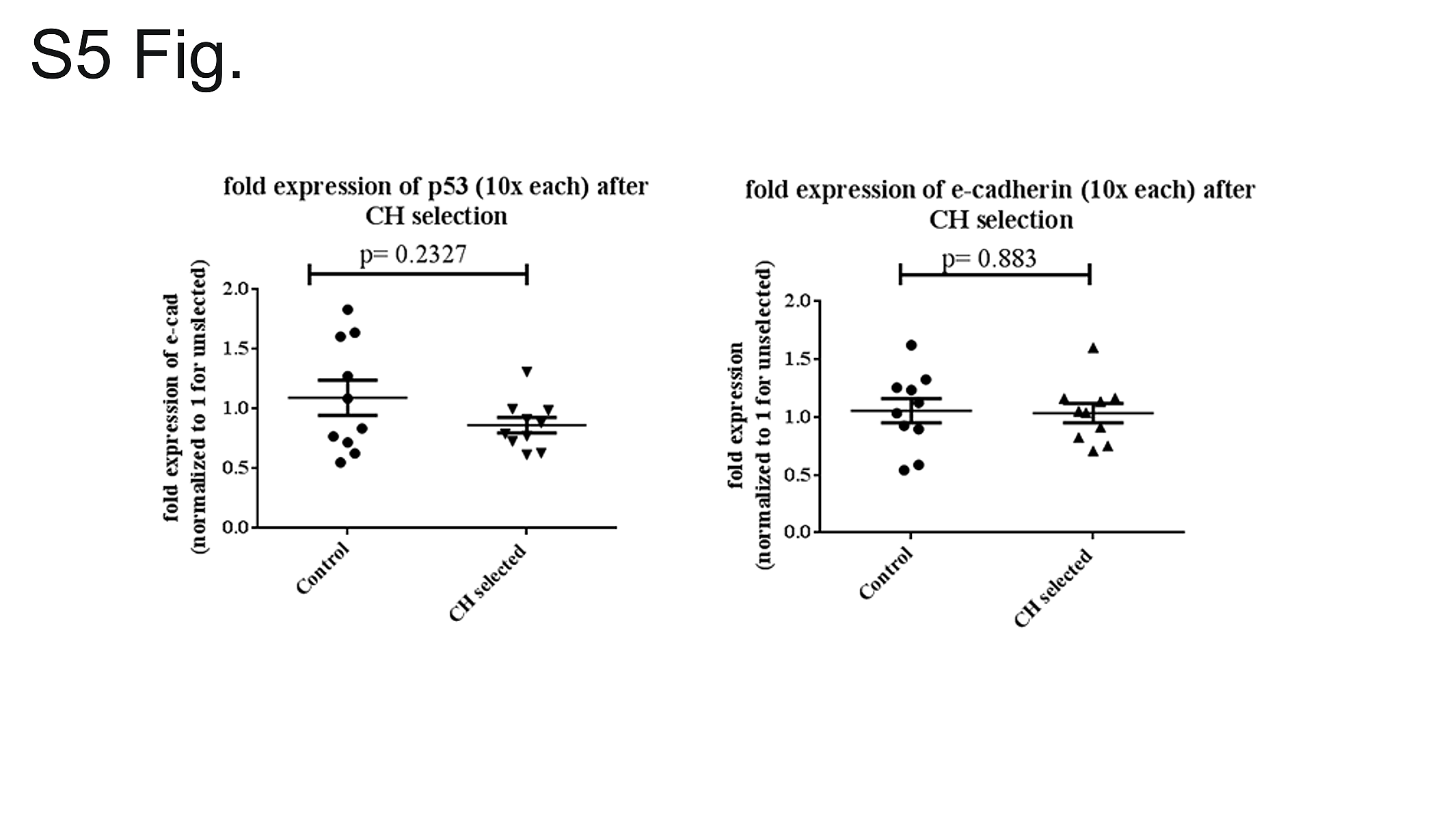

Supplement: S5 Fig — No significant differences in E-cadherin or p53 expression were detected following treatment with chronic hypoxia. RNA was isolated from 10 Chronic Hypoxia treated clones and 10 passaged control clones. mRNA levels of p53, E-cadherin, and actin was measured by quantitative RT-PCR. (TIF) [file pone.0120958.s005.tif]

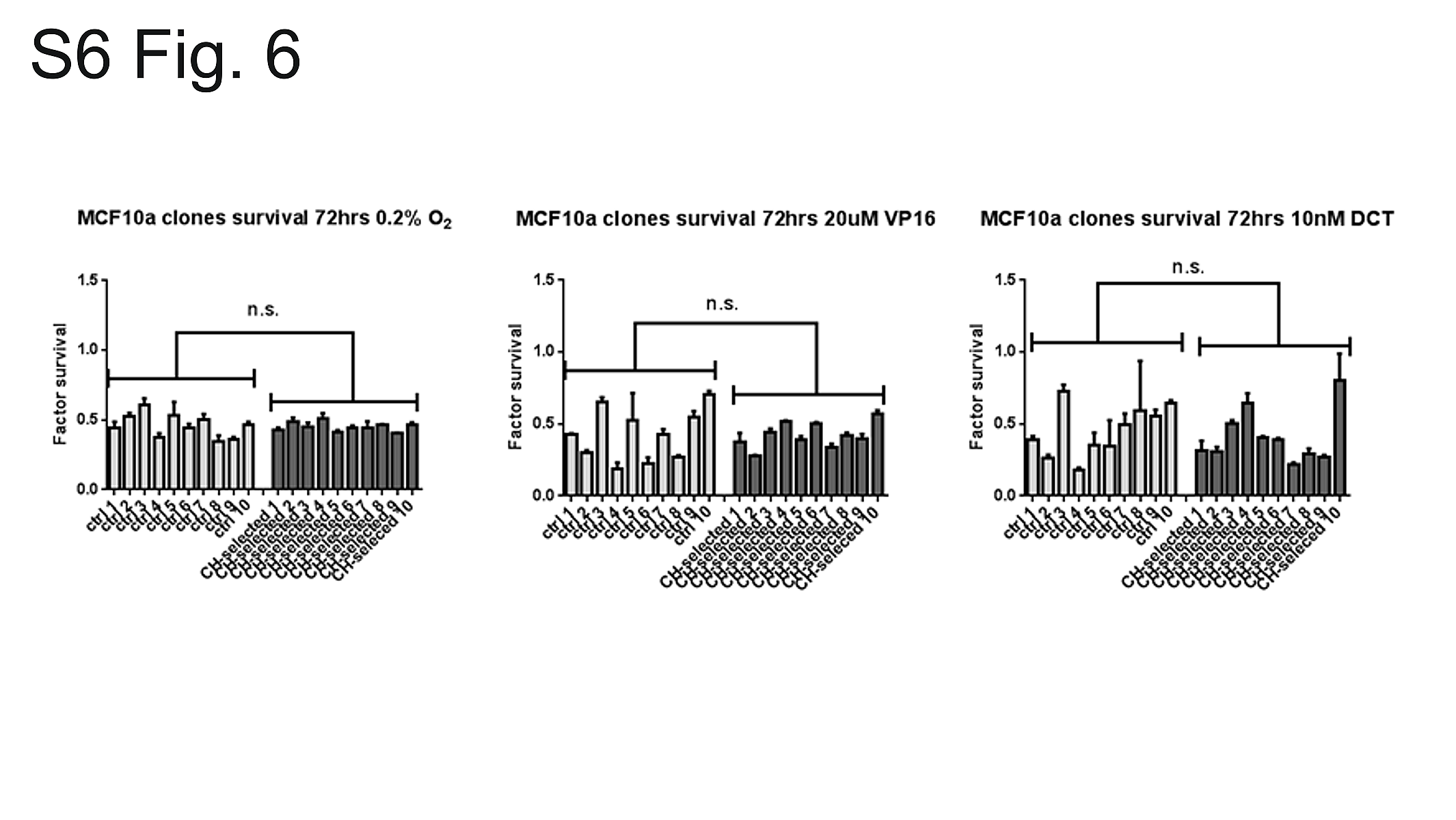

Supplement: S6 Fig — Chronic hypoxia treated clones and control clones were treated with 20μM etoposide, 10nM Docetaxel, 0.2% O2, or untreated in normal growth conditions for 72 hours. No significant difference in survival under any of these conditions was measured. (TIF) [file pone.0120958.s006.tif]

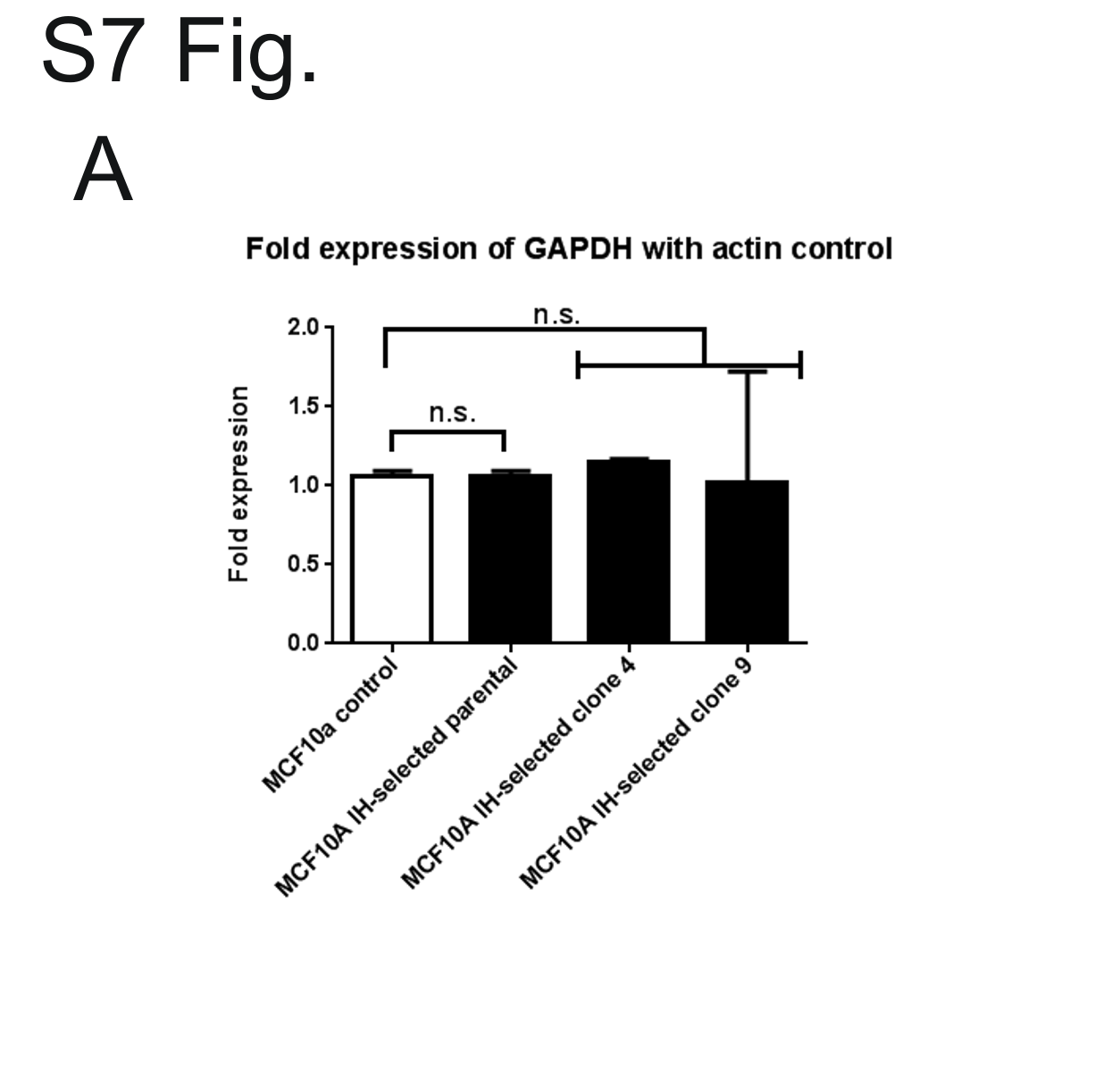

Supplement: S7 Fig — mRNA was isolated from MCF10A parental control cells, MCF10A parental IH-selected cells, and MCF10a IH selected clone 4 and MCF10A IH-selected clone 9. Fold expression of GAPDH over actin as a loading control was measured. No significant difference was measured for MCF10A IH-selected parental cells (p = 0.9400) or IH-selected clones 4 and 9 (p = .9751). (TIF) [file pone.0120958.s007.tif]

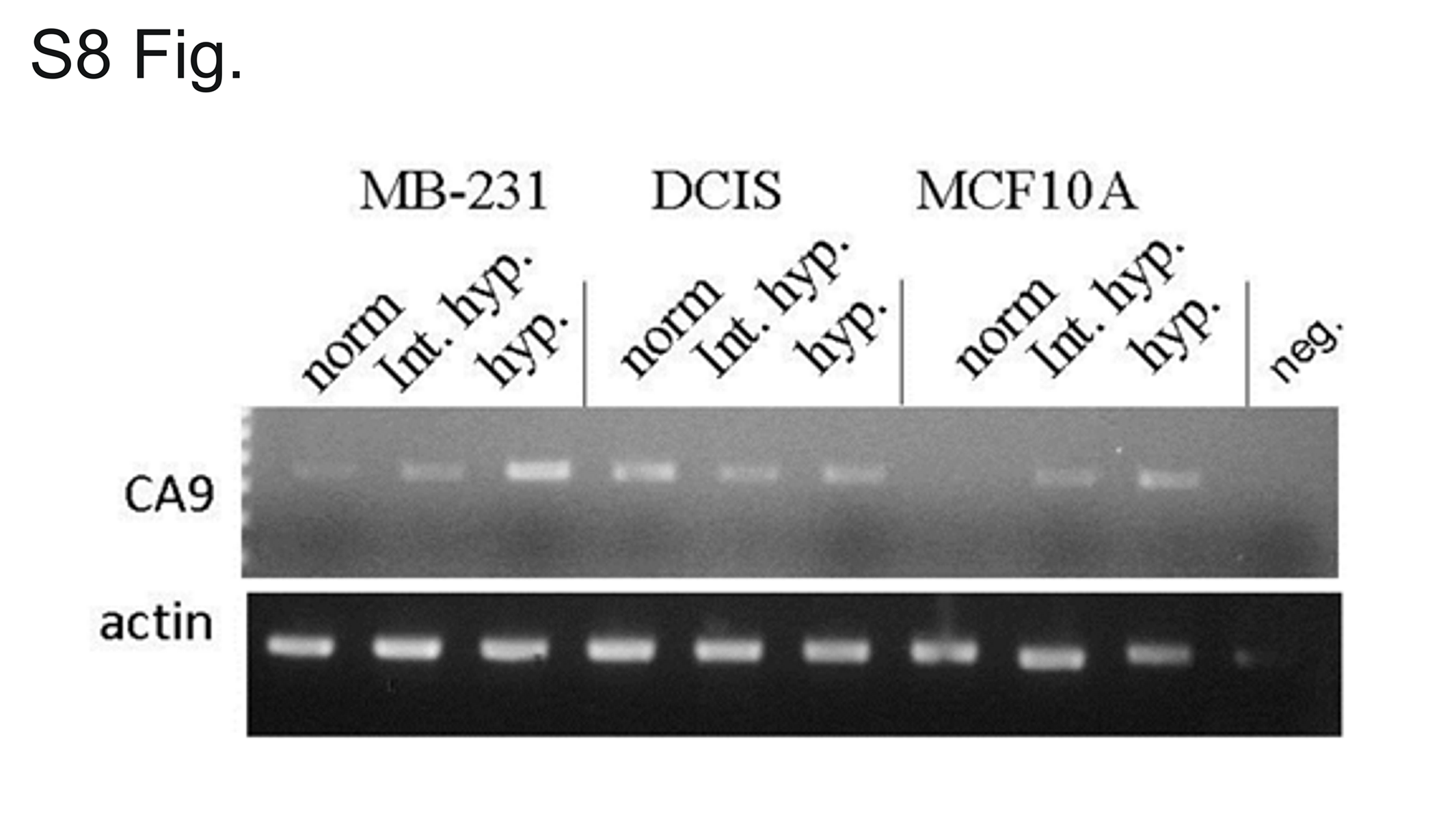

Supplement: S8 Fig — All cells were cultured for 6 days, then RNA was extracted, followed by semi-quantitative PCR to measure CA-IX expression. MDA-MB-231 cells exhibit some constitutive CA-IX expression that is increased under IH and hypoxia. DCIS cells constitutively express about the same amount of CA-IX. MCF10A cells only express CA-IX while under IH or hypoxia. (TIF) [file pone.0120958.s008.tif]

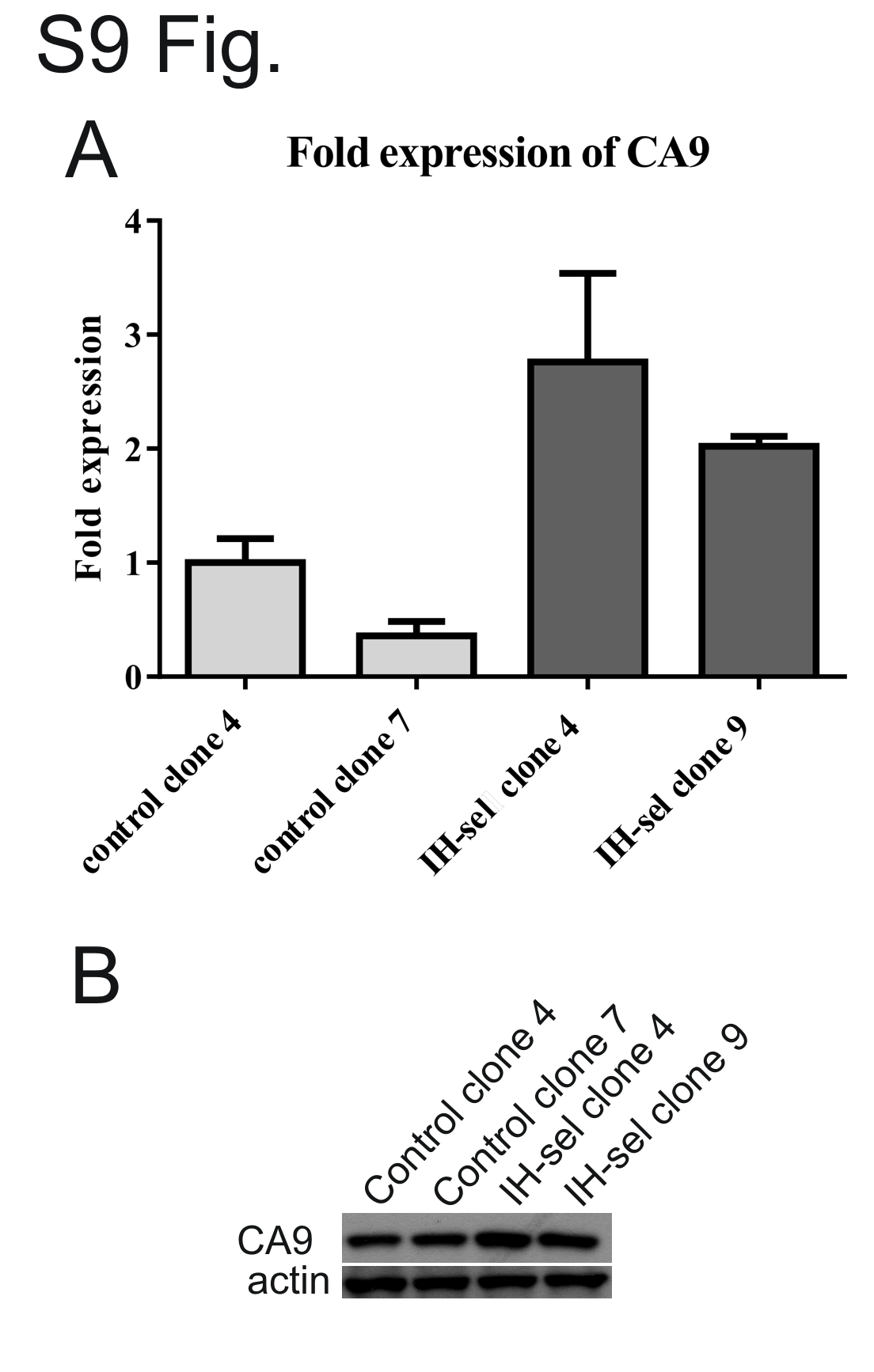

Supplement: S9 Fig — IH-selected clones exhibited an increase in CA-IX expression. Detection of CA-IX by western blot revealed a modest increase following IH-selection. (TIF) [file pone.0120958.s009.tif]

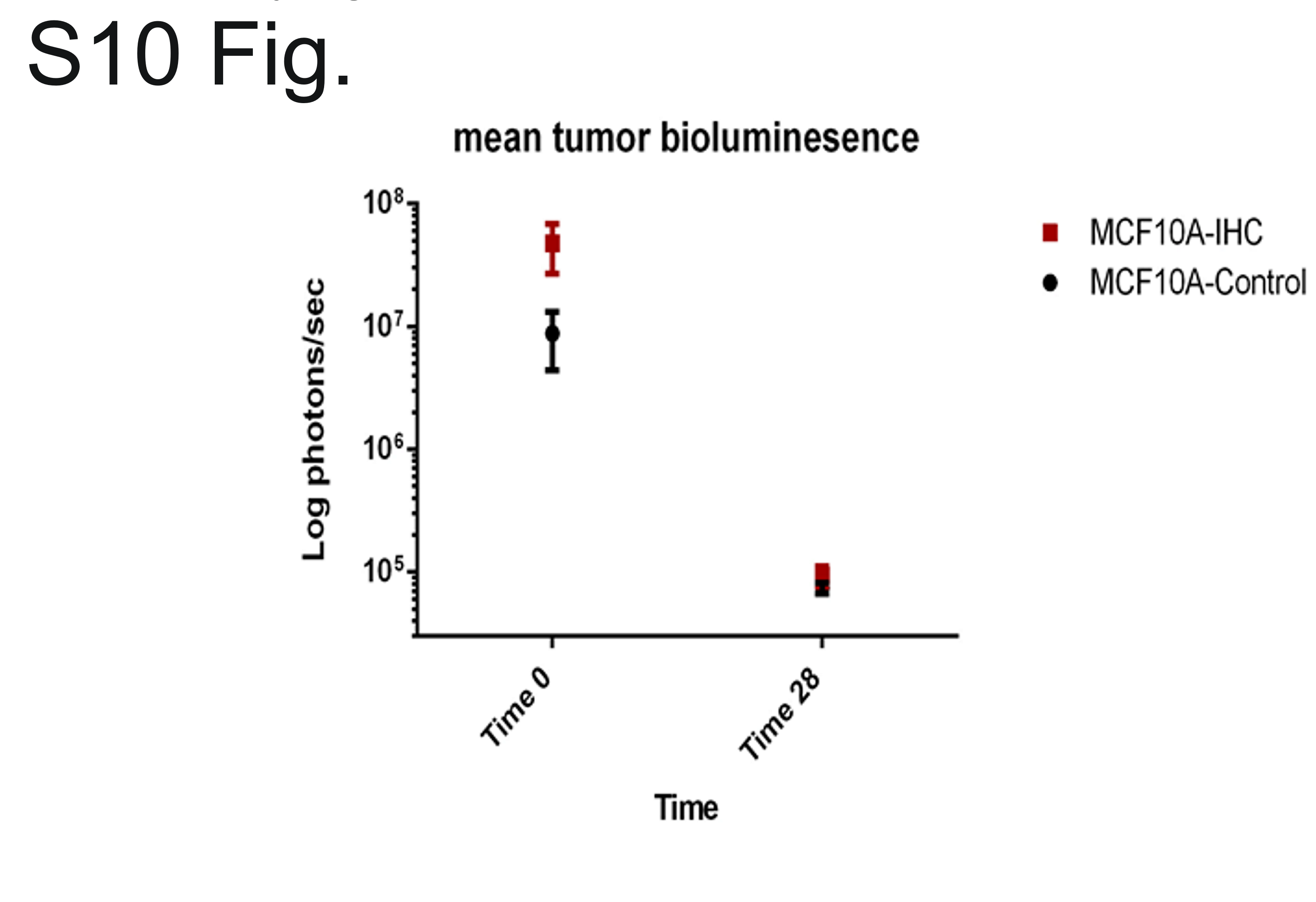

Supplement: S10 Fig — MCF10A-luc control and IH selected cells were injected subcutaneously in to the right and left flank of Nu/Nu male mouse (n = 4) at a concentration of 1–5x10^7^. Mean tumor bioluminescence was quantified in each group indicating no growth difference between the two cohorts at 4 weeks post injections. (TIF) [file pone.0120958.s010.tif]

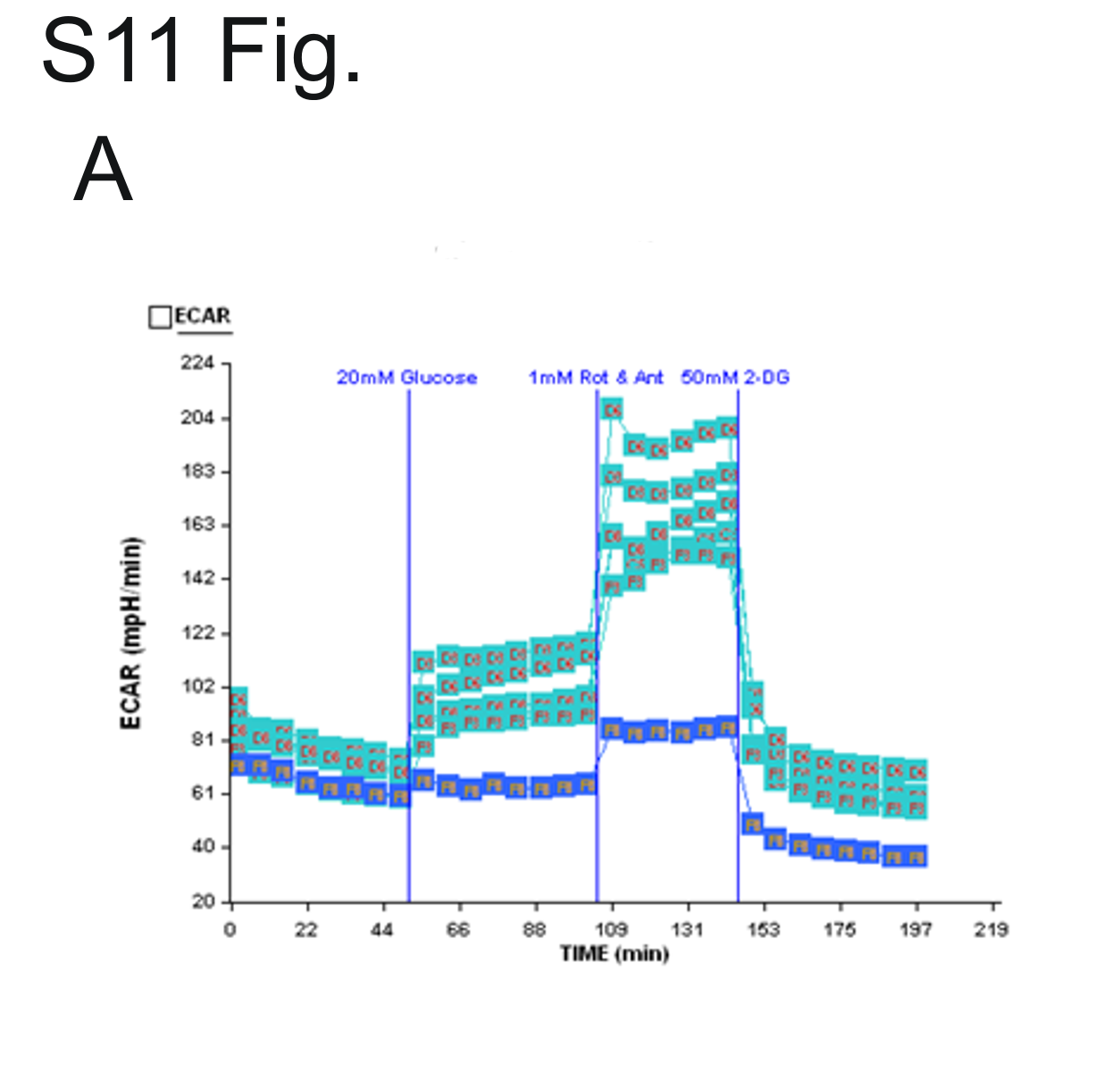

Supplement: S11 Fig — MCF10A cells were subjected to either standard growth conditions (25mM glucose, pH 7.2, 21%O2) or to low glucose, low pH, hypoxic conditions (0.5mM glucose, pH 6.7, 0.2% O2) for two weeks. Metabolic analysis of clones of the low pH low glucose, hypoxic growth cells (teal) exhibited increased glycolysis relative to control cells (blue). (TIF) [file pone.0120958.s011.tif]
